# Genome sequence of walking catfish (*Clarias batrachus*) provides insights into terrestrial adaptation

**DOI:** 10.1186/s12864-018-5355-9

**Published:** 2018-12-20

**Authors:** Ning Li, Lisui Bao, Tao Zhou, Zihao Yuan, Shikai Liu, Rex Dunham, Yuanning Li, Kun Wang, Xiaoyan Xu, Yulin Jin, Qifan Zeng, Sen Gao, Qiang Fu, Yang Liu, Yujia Yang, Qi Li, Axel Meyer, Dongya Gao, Zhanjiang Liu

**Affiliations:** 1 Fish Molecular Genetics and Biotechnology Laboratory, School of Fisheries, Aquaculture and Aquatic Sciences, Auburn University, Auburn, AL 36849 USA; 2 School of Fisheries, Aquaculture and Aquatic Sciences, Auburn University, Auburn, AL 36849 USA; 3 Department of Biological Sciences & Molette Biology Laboratory for Environmental and Climate Change Studies, Auburn University, Auburn, AL 36849 USA; 4 Center for Ecological and Environmental Sciences, Northwestern Polytechnical University, Xi’an, 710072 China; 5 Shellfish Genetics and Breeding Laboratory, Fisheries College, Ocean University of China, Qingdao, 266003 Shandong China; 6 Department of Biology, University of Konstanz, 78464 Konstanz, Germany; 7 Department of Biology, College of Arts and Sciences, Syracuse University, Syracuse, NY 13244 USA

**Keywords:** Fish, Genome, Adaptation, Evolution, Duplication, Air-breathing organ

## Abstract

**Background:**

Walking catfish (*Clarias batrachus*) is a freshwater fish capable of air-breathing and locomotion on land. It usually inhabits various low-oxygen habitats, burrows inside the mudflat, and sometimes “walks” to search for suitable environments during summer. It has evolved accessory air-breathing organs for respiring air and corresponding mechanisms to survive in such challenging environments. Thereby, it serves as a great model for understanding adaptations to terrestrial life.

**Results:**

Comparative genomics with channel catfish (*Ictalurus punctatus*) revealed specific adaptations of *C. batrachus* in DNA repair, enzyme activator activity, and small GTPase regulator activity. Comparative analysis with 11 non-air-breathing fish species suggested adaptive evolution in gene expression and nitrogenous waste metabolic processes. Further, myoglobin, olfactory receptor related to class A G protein-coupled receptor 1, and sulfotransferase 6b1 genes were found to be expanded in the air-breathing walking catfish genome, with 15, 15, and 12 copies, respectively, compared to non-air-breathing fishes that possess only 1–2 copies of these genes. Additionally, we sequenced and compared the transcriptomes of the gill and the air-breathing organ to characterize the mechanism of aerial respiration involved in elastic fiber formation, oxygen binding and transport, angiogenesis, ion homeostasis and acid-base balance. The hemoglobin genes were expressed dramatically higher in the air-breathing organ than in the gill of walking catfish.

**Conclusions:**

This study provides an important genomic resource for understanding the adaptive mechanisms of walking catfish to terrestrial environments. It is possible that the coupling of enhanced abilities for oxygen storage and oxygen transport through genomic expansion of myoglobin genes and transcriptomic up-regulation of hemoglobin and angiogenesis-related genes are important components of the molecular basis for adaptation of this aquatic species to terrestrial life.

**Electronic supplementary material:**

The online version of this article (10.1186/s12864-018-5355-9) contains supplementary material, which is available to authorized users.

## Background

The walking catfish (*Clarias batrachus*) is a freshwater teleost species with air-breathing capability native to Southeast Asia, where it is widely used as an aquaculture species due to its high economic value as food [1]. Unfortunately, this species also is categorized as endangered because of over-exploitation and habitat alterations in its native India and Bangladesh [2–4]. On the other hand, it is an invasive species in the United States, currently found in over ten states on the eastern and western coasts (http://maps.iucnredlist.org/map.html?id=166613), but with established populations likely only in Florida [5]. *C. batrachus* was imported into Florida from Thailand in the early 1960s [6]. It has been thought to be damaging to native fish populations; however, there is little evidence to support this except that they do invade aquaculture facilities and can cause severe damage to cultured fish populations (Florida Museum, University of Florida 2017; https://www.floridamuseum.ufl.edu/fish/discover/species-profiles/clarias-batrachus/). Their air breathing capability allows them to disperse quickly across terrestrial environments, a feature that most native fish do not have.

A combination of traits - such as high fecundity, adaptation to adverse ecological conditions, and in particular the ability to “walk” between isolated water bodies - make this fish an especially successful invasive species. It is able to inhabit various low-oxygen habitats such as swamps and wetlands, and burrows inside the mudflat during summer periods [3, 7, 8]. When the original habitat dries up or after a heavy rainfall, the walking catfish can make snake-like movements to move from one body of water to another by pulling its body across land with the pectoral fins [3, 8, 9]. The accessory air-breathing organ is another key innovation to survival during its terrestrial walk to the next aquatic environment. This structure is derived from the gill, and the air-breathing organ in particular consists of suprabranchial chambers, gill fans and arborescent organs [10, 11]. *Clarias* can breathe air as well as using gills for respiration in water [8]. Its “walking” abilities allow *Clarias* to cope with respiration challenges without a lung in the terrestrial environment, as well as adaptation to extreme environmental challenges such as high ammonia as well as hypoxic and desiccation stresses [12]. This makes *C. batrachus* a perfect model for studying the evolution of adaptations such as terrestrial dispersal, aerial respiration and high tolerance to hypoxia and ammonia.

Recent genome projects have demonstrated that comparative genomic analysis combined with transcriptomic analysis allow the elucidation of the genomic basis for adaptation to terrestrial life in mangrove rivulus (*Kryptolebias marmoratus*) and mudskippers (*Bolelphthalmus pectinirostris*, *Scartelaos histophorus*, *Periophthalmodon schlosseri* and *Periophthalmus magnuspinnatus*) [13, 14]. Mangrove rivulus mainly utilizes its skin and mudskippers mainly utilize their buccal cavity to breathe air [15, 16], while *C. batrachus* utilizes an accessory air-breathing organ. It is of great interest to determine the genomic basis of adaptations of aquatic species to the terrestrial environment based on the genome sequence of *C. batrachus* and the characterization of some of its genomic features that are potentially linked to terrestrial adaptations.

## Results

### Genome assembly and annotation

The statistics for the draft genome sequence assembly are shown in Table 1. The final assembly contained 10,041 scaffolds, with a scaffold N50 of 361.2 kb. The assembly covered a total of 821 Mb, similar to the genome size of 854 Mb estimated from ALLPATHS-LG, but slightly smaller than the estimated 900 Mb based on the Feulgen densitometry method [17] and the 1.17 Gb based on the bulk fluorometric assay method [18].

**Table 1:** Summary statistics for walking catfish (*Clarias batrachus*) genome sequencing, assembly and annotation

Genome sequencing				
Library	# of reads	Read length	Trimmed data	Genome coverage
Paired-end 180 bp	426 M	100 bp	39.2 Gb	46X
Mate-pair 3 kb	483 M	100 bp	32.1 Gb	38X
Genome assembly				
	Total	N50	Longest	Assembled size
Contigs	87,962	19.0 kb	194 kb	747.7 Mb
Scaffolds	10,041	361.2 kb	2843 kb	821.8 Mb
Genome annotation				
Number of genes	Repetitive elements content			
22,914	30.3%			

The completeness of the genome assembly was assessed by mapping the 248 core eukaryotic genes (CEGs) from CEGMA v2.5 [19] to the genome sequence. The draft genome sequence covered 95.2% of the CEGs (Additional file 1: Table S1). When the 3023 genes from vertebrate BUSCO orthologues [20] were mapped to the genome assembly, the draft genome sequence included 83.9% of these genes (Additional file 1: Table S1). As our objective was to identify additional gene copies or novel genes in the walking catfish not found in non-air-breathing fishes, which may account for its adaptations for partial living on land, this level of completeness is reasonable, although a small percentage of missing genes may reduce the capacity of identifying more such genes. The assembly was assessed also to be accurate. The 5 longest scaffolds (1.3 Mb–2.2 Mb) assembled using a second software, ABySS, had 99.4% alignments with the genome sequence assembled using ALLPATHS-LG (Additional file 1: Table S2).

**Fig. 1:**
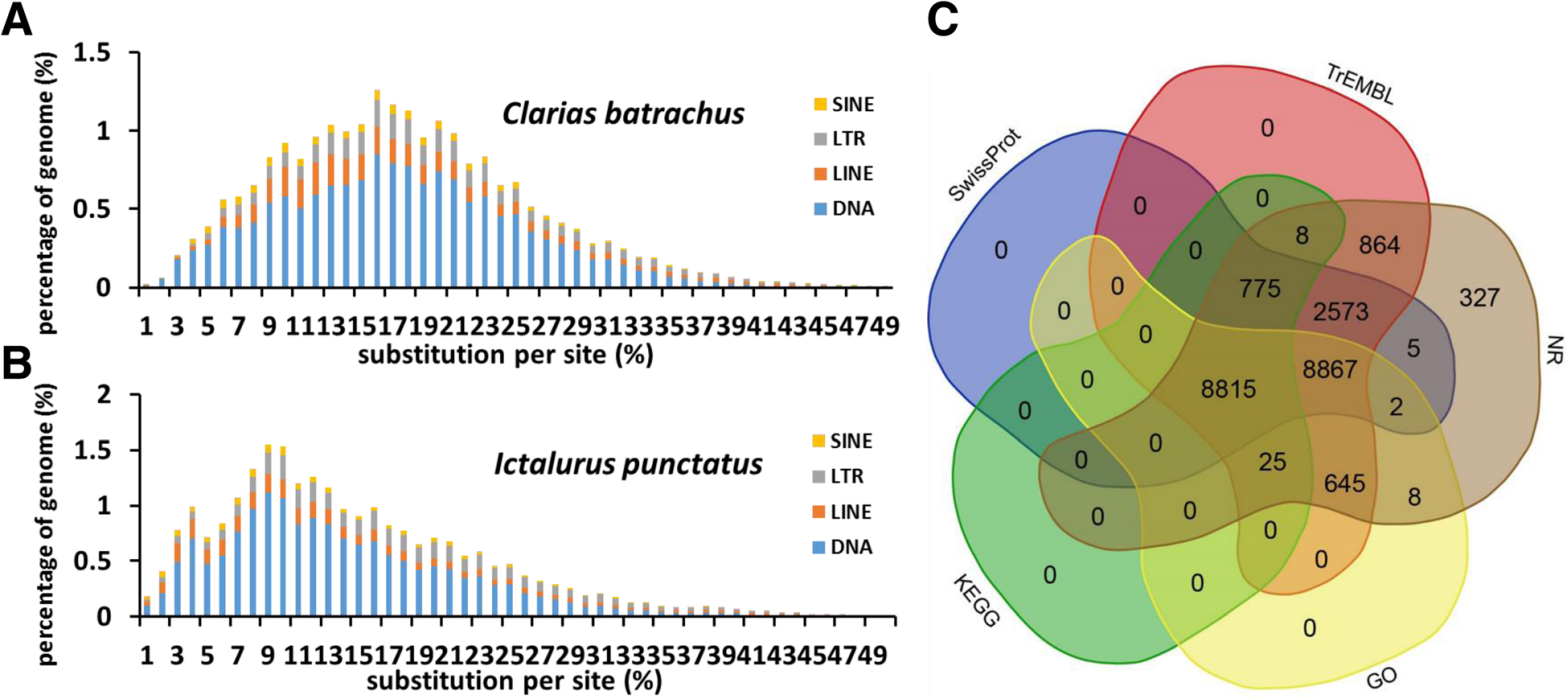
Annotation of the walking catfish genome. The distribution of repetitive elements and their contents are shown for *Clarias batrachus* (**a**) and *Ictalurus punctatus* (**b**). The average number of substitutions per site for each fragment was estimated using the Jukes-Cantor formula. SINE, short interspersed elements; LTR, long terminal repeats; LINE, long interspersed elements. **c** Venn diagram showing the number of homologues of the predicted genes from the *C. batrachus* genome in various databases: NR (non-redundant protein database), TrEMBL, and SwissProt

The *C. batrachus* genome had a GC content of 39.2%, similar to those of other fish species [21, 22]. Repetitive elements comprised 30.3% of the genome (Table 1, Additional file 1: Table S3). Although the contents of repetitive elements in the *C. batrachus* genome were similar to those in the channel catfish genome [23], it appeared that the number of substitutions per site for *C. batrachus* repetitive elements exhibited a peak at about 16% (Fig. 1a), higher than that of channel catfish repetitive elements (~ 10%, Fig. 1b), indicating that its repetitive elements had a longer evolutionary history and/or have been more active through evolution than those of the channel catfish genome [24].

A total of 22,914 genes were annotated from the *C. batrachus* genome sequence, of which 19,834 genes (86.6%) were supported by RNA-Seq evidence from the gill and the air-breathing organ. Among the identified protein-coding genes, the majority (22,587, 98.6%) were supported by matches from at least two publicly available databases including the non-redundant protein database, SwissProt and TrEMBL subsets of the UniProt database [25], KEGG and GO terms (Fig. 1c).

### Comparative genomic analysis

**Fig. 2:**
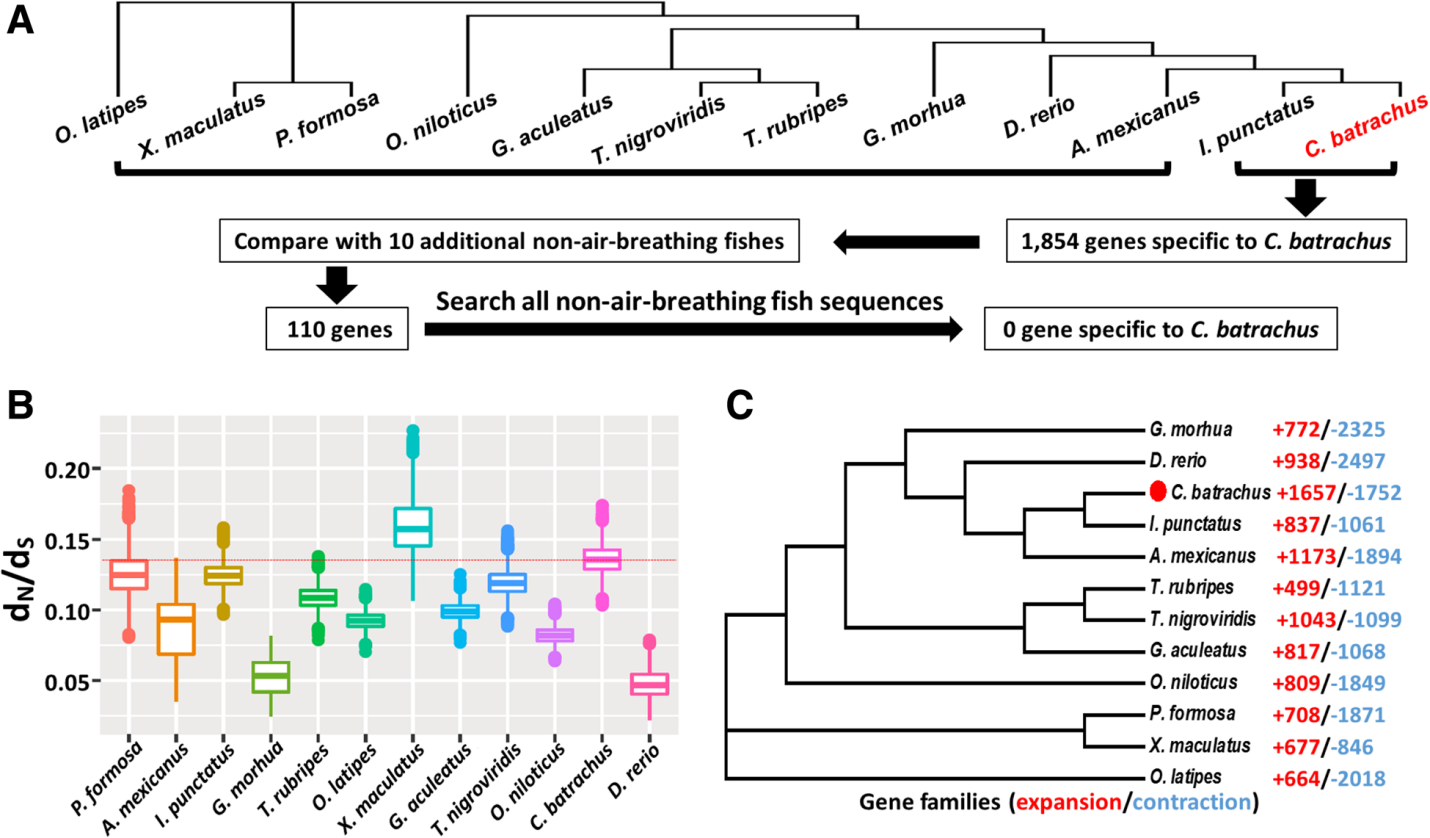
Comparisons of genomic features of *Clarias batrachus* with those non-air-breathing fish species. **a** Schematic presentation of comparative genomic analyses of *C. batrachus* against non-air-breathing teleost fishes. In the catfish lineage, the protein sequences of the *C. batrachus* and *I. punctatus* genomes were comparatively subtracted, resulting in the identification of 1854 genes specific to the *C. batrachus* genome; similarly, 10 additional non-air-breathing fishes were added to compare with *C. batrachus* genome, resulting in the identification of 110 genes that were only present in the *C. batrachus* genome. The names of these 110 *C. batrachus* specific genes were searched with all existing sequences from non-air-breathing fish species in the NCBI database, resulting in no genes specific to the *C. batrachus* genome. **b** Comparison of the values of d_N_/d_S_ ratio among various fish species against the ancestor estimated from 150 randomly picked single-copy genes with 10,000 bootstrap replicates. The red line represents the average d_N_/d_S_ value in *C. batrachus*, noting that it is the second most rapidly evolving genome. **c** The number of gene families exhibiting expansion (red) / contraction (blue). *C. batrachus* is marked with red solid circle, showing that it has the largest number of expanded gene families. *D. rerio*, *Danio rerio*; *G. aculeatus*, *Gasterosteus aculeatus*; *T. nigroviridis*, *Tetraodon nigroviridis*; *T. rubripes*, *Takifugu rubripes*; *O. latipes*, *Oryzias latipes*; *G. morhua*, *Gadus morhua*; *A. mexicanus*, *Astyanax mexicanus*; *O. niloticus*, *Oreochromis niloticus*; *X. maculatus*, *Xiphophorus maculatus*; *P. formosa*, *Poecilia formosa*; *I. punctatus*, *Ictalurus punctatus*

To identify genes that are specific to the *C. batrachus* genome, we first compared the genes between the walking catfish and channel catfish (Fig. 2a, detailed methods described in the “Methods”). They both belong to Order Siluriformes, and therefore shared the highest number of orthogroups compared to other fish species in this study (Additional file 1: Table S4), but the walking catfish possesses the air-breathing organ while the channel catfish does not. A total of 1854 genes were present in the walking catfish, but absent from channel catfish (Additional file 1: Table S5). These genes were enriched for “DNA repair”, “enzyme activator activity” and “small GTPase regulator activity” (Additional file 1: Table S6), which may be associated with its adaptation to the terrestrial life, such as responding to increased DNA damage and accelerated metabolic processes. Small GTPases are well-known for maintaining cell adhesion, cell migration, gene transcription and cytogenesis [26, 27], and one of their critical modulators, namely “guanyl-nucleotide exchange factor activity”, was also found to be significantly enriched (Additional file 1: Table S6). Furthermore, small GTPases were also reported to be under selection in the alkaline-tolerant population compared with the flowing freshwater population of Amur ide *Leuciscus waleckii*, reflecting their roles in regulating ion transport and acid-base balance under extreme environmental conditions [28].

To further narrow down the list of genes potentially present in the walking catfish but absent in non-air-breathing fishes, the status of the 1854 genes were determined in 10 additional non-air-breathing fish species (Fig. 2a). Only 110 genes were then found to be present in the walking catfish, but absent in the 10 non-air-breathing fishes. When this list of genes was further investigated by comparison with sequences from all non-air-breathing fish species in the NCBI databases, no genes were found to be specific to the walking catfish (Fig. 2a). Although it is possible that the genome sequence assembly is incomplete and that unique and specific genes of the walking catfish could have been missed, it is unlikely that the walking catfish harbors a large number of specific genes for air-breathing.

Next, we also determined the ratio of non-synonymous substitutions to synonymous substitutions using 705 single-copy orthologous genes in the *C. batrachus* and the other 11 non-air-breathing fishes. As shown in Fig. 2b, *C. batrachus* apparently evolved rapidly with the second highest d_N_/d_S_ ratio, next only to *X. maculatus*, suggesting that parts of its genome are under strong selection. Of the 705 single-copy genes, 132 were positively selected (Additional file 1: Table S7). These genes were mainly enriched in “mitochondrial intermembrane space”, “nucleoplasm part”, “RNA polymerase II transcription factor complex” and “nuclear DNA-directed RNA polymerase complex” (Additional file 1: Table S8), indicating the accelerated evolution of genes involved in regulation of gene expression in *C. batrachus*. The overrepresented pathway “gene expression” included a list of genes related to transcription factors (*med6*, *med14*, *gtf2e2*, *mnat1* and *nfyc*), RNA binding protein (*paip1*), mRNA splicing factors (*cstf2*, *sf3b2*, *rbm8a* and *cpsf5*), chromatin binding (*noc2l*) and translation initiation factor (*eif3m*). Additionally, the GO term “cellular nitrogen compound metabolic process” was also found to be enriched for genes under positive selection (Additional file 1: Table S8).

### Gene expansion

In addition to analysis of positive selection on single-copy genes, levels of gene family dynamics, including expansion due to gene duplication and contraction due to gene loss, were studied. The *C. batrachus* genome shows signs of expansion in 1657 families and contraction in 1752 families (Fig. 2c). Among the 12 studied fish genomes, it had the largest number of expanded gene families, suggesting that its adaptation to terrestrial lifestyle may have been mediated partly by gene family expansion. Among the 1657 expanded gene families, three families were significantly expanded (*P*-value = 0) in *C. batrachus*: myoglobin (*mb*), olfactory receptor related to class A G protein-coupled receptor 1 (*ora1*) and sulfotransferase 6b1 (*sult6b1*).

**Fig. 3:**
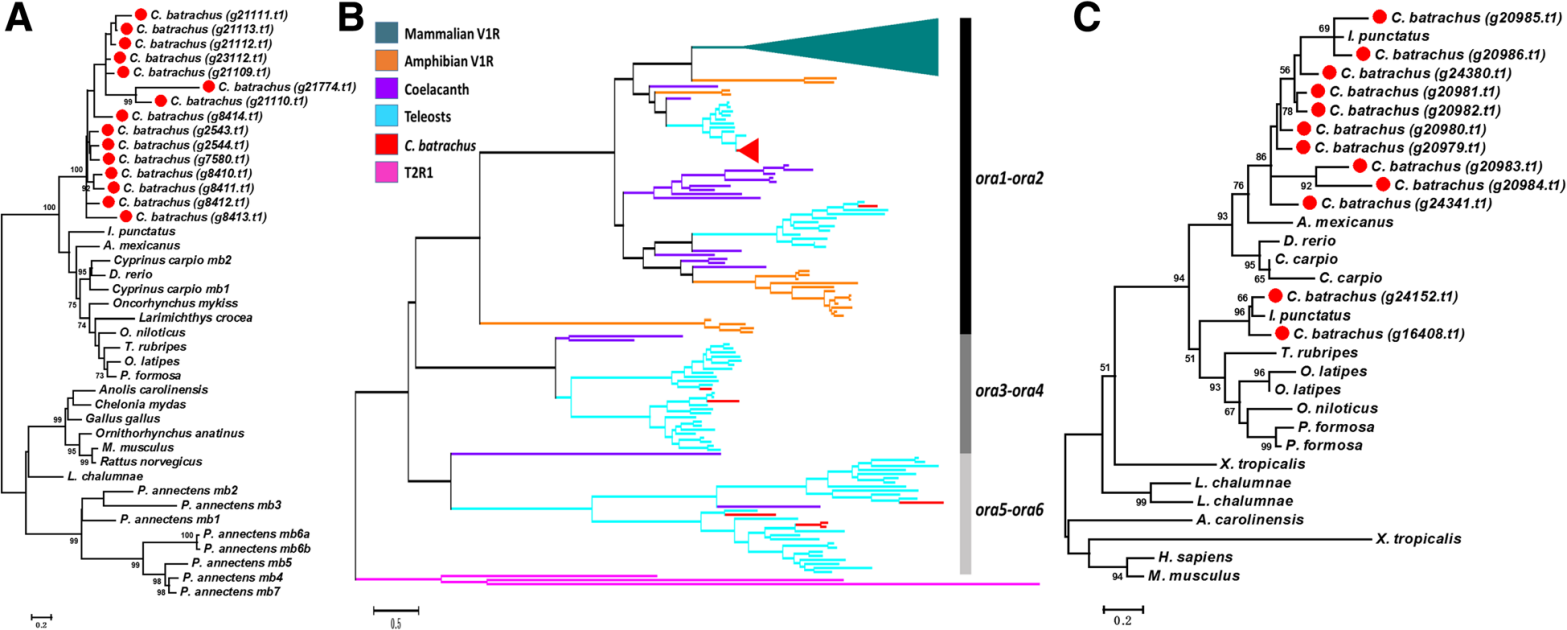
Maximum likelihood phylogenetic trees of expanded gene families in the *Clarias batrachus* genome. **a** Phylogenetic tree of myoglobin (*mb*) in vertebrates showing gene expansion of *mb* in the *C. batrachus* genome. The red solid circles represent the genes in the *C. batrachus* genome. Bootstrap support values (1000 replications) are indicated on the nodes. **b** Phylogenetic tree of the olfactory receptor related to class A G protein-coupled receptor (*ora*) gene family in vertebrates showing the expansion of *ora1* in the *C. batrachus* genome with taste receptor of type 2 member 1 (T2R1) as an outgroup. The three clades (*ora1*-*ora2*, *ora3*-*ora4* and *ora5*-*ora6*) formed from six members are indicated on the right of the figure. The dark green solid triangle represents the expansion of vomeronasal type 1 receptors (V1Rs) in mammals. The red solid triangle represents the 15 copies in the *C. batrachus* genome. The term “teleosts” here is used to indicate the non-air-breathing fish species discussed in this study. The detailed phylogenetic tree with species names and sequence names is displayed in the Additional file 1: Figure S2. **c** Phylogenetic tree of sulfotransferase 6b1 (*sult6b1*) in vertebrates showing gene expansion of *sult6b1* in the *C. batrachus* genome. The red solid circles represent the genes in the *C. batrachus* genome. Bootstrap support values (1000 replications) are indicated on the nodes. *D. rerio*, *Danio rerio*; *G. aculeatus*, *Gasterosteus aculeatus*; *T. nigroviridis*, *Tetraodon nigroviridis*; *T. rubripes*, *Takifugu rubripes*; *O. latipes*, *Oryzias latipes*; *G. morhua*, *Gadus morhua*; *A. mexicanus*, *Astyanax mexicanus*; *O. niloticus*, *Oreochromis niloticus*; *X. maculatus*, *Xiphophorus maculatus*; *P. formosa*, *Poecilia formosa*; *I. punctatus*, *Ictalurus punctatus*; *C. carpio*, *Cyprinus carpio*; *P. annectens*, *Protopterus annectens*; *M. musculus*, *Mus musculus*; *H. sapiens*, *Homo sapiens*; *X. tropicalis*, *Xenopus tropicalis*; *L. chalumnae*, *Latimeria chalumnae*; *A. carolinensis*, *Anolis carolinensis*

The myoglobin gene exists in almost all vertebrate species with one-to-two copies in the genome, except for the seven copies in the West African lungfish (*Protopterus annectens,* obviously air-breathing) genome [29–35]. We found a huge expansion of myoglobin, fifteen copies of the gene, in the *C. batrachus* genome (Fig. 3a, Additional file 1: Table S9). Multiple sequence alignments showed some diversities among them (Additional file 1: Figure S1). These 15 copies of the myoglobin gene were located on six scaffolds. We do not have information of their chromosomal locations, but the sequence analysis indicated that tandem duplications exist in three of the six scaffolds. Based on the flanking genes and syntenic analysis, the fifteen myoglobin genes of *C. batrachus* may be located on different chromosomes. The *ora1* gene also was found to be significantly expanded in the *C. batrachus* genome with 15 copies, while there is only a single copy in most teleost species (Fig. 3b, Additional file 1: Figure S2, Additional file 1: Table S9). The 15 copies of *ora1* genes in *C. batrachus* were found on the same scaffold, suggesting tandem duplications, and these fifteen genes displayed high sequence similarities (Additional file 1: Figure S3). The *sult6b1* gene also was found to be highly expanded in *C. batrachus* with twelve copies, as compared to 1–2 copies in non-air-breathing teleost fishes (Fig. 3c, Additional file 1: Table S9).

### Expression of significantly expanded gene families

The expression of the various copies of the *mb* and *sult6b1* genes in *C. batrachus* was analyzed using RNA-Seq datasets (Table 2). All 15 myoglobin genes were expressed, but in different tissues and at different levels. The brain had an overall high level of expression, consistent with the results in common carp and West African lungfish [32, 35]. The expression levels in the air-breathing organ are of particular interest: two copies of the myoglobin gene, g21774.t1 and g8414.t1, were expressed at high levels, at least 10 times higher than other copies. In all the other tissues, only one copy, g8414.t1, was expressed at very high levels. Interestingly, synteny analyses indicated that g8414.t1 is orthologous to the single-copy myoglobin gene in other fish species (Additional file 1: Figure S4). Its high expression in all tissues suggested that its function may be conserved in evolution. We suggest that these gene copies may be evolved as inducible genes to cope with hypoxic environments such as the terrestrial condition, and thus are related to the adaptation to the transition from water to land. For the *sult6b1* gene, all 12 copies were expressed, but with some tissue-specificity. For instance, g20983.t1 and g24341.t1 were expressed only in the gill, g24152.t1 and g16408.t1 were expressed only in the gill and the air-breathing organ, and g24380.t1 was expressed only in the gill and liver. All 12 copies were expressed in the gill, but the most highly expressed copies were g20980.t1, g20985.t1 and g20986.t1. Notably, all these highly expressed copies were tandem duplicates on the same scaffold (Table 2). The o*ra1* gene is known to be tissue-specific and expressed only in the olfactory epithelium in other fish species [36]. In regards to the five tissues examined with RNA-Seq, no transcripts of *ora1* were detected, consistent with the tissue-specific expression of this gene only in olfactory epithelium in other fish species [36].

**Table 2:** Expression of myoglobin and sulfotransferase 6b1 genes (FPKM) in the air-breathing organ, gill, brain, head kidney and liver of *Clarias batrachus* as determined by analysis of RNA-Seq datasets. RNA-Seq datasets from air-breathing organ and gill were generated from this project; those from brain (SRR2057993), head kidney (SRR2057995), and liver (SRR2052655) were downloaded from NCBI. Each continuous bold and italic gene cluster indicated tandemly duplicated genes. FPKM, Fragments Per Kilobase per Million mapped fragments

Gene name	Gene ID	Air-breathing organ	Gill	Brain	Head kidney	Liver
Myoglobin	***g21109.t1***	0	0	0	2.91	2.84
	***g21110.t1***	0	0	8.48	0.85	4.14
	***g21111.t1***	0.25	0.18	3.14	1.57	1.53
	***g21112.t1***	0.59	0.05	1.45	1.55	0
	***g21113.t1***	0.09	0	4.48	9.88	2.92
	g21774.t1	4.52	5.07	0	1.02	0
	g23112.t1	0.46	0.76	0	0.58	0
	***g2543.t1***	0	0	26.84	25.61	5.11
	***g2544.t1***	0	0	15.71	12.23	3.31
	g7580.t1	0	0	0	2.32	0
	***g8410.t1***	0	0	17.4	23.41	3.31
	***g8411.t1***	0	0	0	0.59	0
	***g8412.t1***	0	0.56	16.23	13.52	3.27
	***g8413.t1***	0	0.16	0	0	0
	***g8414.t1***	6.18	139.88	838.66	643.35	145
Sulfotransferase 6b1	g16408.t1	0.2	1.16	0	0	0
	***g20979.t1***	2.78	1.22	3.96	1.94	1.95
	***g20980.t1***	23.9	28.5	13.36	18.93	2.09
	***g20981.t1***	3.42	3.79	6.16	3.34	1.28
	***g20982.t1***	2.91	2.42	1.57	1.62	1.28
	***g20983.t1***	0	0.58	0	0	0
	***g20984.t1***	0.54	0.53	0.57	1.43	1.75
	***g20985.t1***	9.37	16.57	5.6	4.94	0.26
	***g20986.t1***	24.61	14.81	89.49	44.81	0
	g24152.t1	0.13	0.53	0	0	0
	g24341.t1	0	0.06	0	0	0
	g24380.t1	0	0.31	0	0	15.45

### Comparative transcriptomic analysis between gill and the air-breathing organ

To understand the transcriptomic differences between the gill and the air-breathing organ, we sequenced the transcriptomes of the gill and the air-breathing organ (Additional file 1: Table S10), analyzed the expression levels of all transcripts (Additional file 1: Table S11), and determined the differentially expressed genes (DEGs, Additional file 1: Table S12). A total of 813 DEGs were identified between the gill and the air-breathing organ, of which 335 and 478 genes were up-regulated and down-regulated in the air-breathing organ, respectively. These results were validated by determining the expression levels of a fraction of these genes using qRT-PCR (Additional file 1: Figure S5). All the DEGs were subjected to GO and pathway enrichment analyses, and the significantly over-represented GO terms and pathways in the DEGs are listed in Additional file 1: Table S13 and Additional file 1: Table S14, respectively. As summarized in Table 3, a total of 51 genes belonging to five functional groups were highly and differentially expressed between the gill and the air-breathing organ.

**Table 3:** A list of genes differentially expressed between the gill and the air-breathing organ in *Clarias batrachus*

Category name	Gene name	Gene ID	FDR
Acid-base balance	Solute carrier family 4 member 1	g12078.t1	0
	Carbonic anhydrase	g8816.t1	0
	Carbonic anhydrase 2	g3019.t1	1.60E-07
	Carbonic anhydrase 4	g7074.t1	7.21E-07
	Carbonic anhydrase 9	g411.t1	5.37E-05
	Carbonic anhydrase 6	g456.t1	7.39E-04
Ion homeostasis	Sodium/potassium-transporting ATPase subunit alpha-1	g15272.t1	0
	Sarcoplasmic/endoplasmic reticulum calcium ATPase 1	g16367.t1	0
	Sarcoplasmic/endoplasmic reticulum calcium ATPase 1	g5688.t1	0
	Sarcoplasmic/endoplasmic reticulum calcium ATPase 2	g9634.t1	0
	Sodium potassium-transporting ATPase subunit beta-233	g9311.t1	0
	Adenosylhomocysteinase 3	g1970.t1	2.72E-10
	Adenosylhomocysteinase 3	g1011.t1	1.19E-08
	Calsequestrin-1	g3495.t1	5.23E-04
	Calsequestrin-2	g9400.t1	3.67E-02
	Pendrin	g2970.t1	0
	Sodium/hydrogen exchanger 3	g3908.t1	0
Elastic fiber formation	Transforming growth factor beta-2	g2430.t1	8.22E-03
	Fibulin-1	g4468.t1	9.66E-08
	Transforming growth factor beta-3	g14668.t1	2.68E-02
	Fibulin-2	g20129.t1	5.73E-03
	Bone morphogenetic protein 4	g189.t1	8.79E-06
	Fibronectin	g6260.t1	0
	Fibronectin	g12205.t1	9.01E-03
	Latent-transforming growth factor beta-binding protein 3	g14988.t1	8.22E-03
Oxygen binding and transport	Hemoglobin subunit alpha	g20835.t1	0
	Hemoglobin subunit beta	g20836.t1	3.55E-11
	Hemoglobin subunit alpha	g20837.t1	0
	Hemoglobin subunit beta	g20838.t1	0
	Hemoglobin subunit beta	g21168.t1	0
	Hemoglobin subunit alpha	g21169.t1	0
	Hemoglobin subunit beta	g21170.t1	0
	Hemoglobin subunit alpha	g21171.t1	0
Angiogenesis	Sphingosine 1-phosphate receptor 1	g5232.t1	7.89E-03
	Fibronectin	g6260.t1	8.79E-06
	Semaphorin-3E	g5331.t1	6.86E-05
	C-X-C chemokine receptor type 4	g23603.t1	0
	Neuropilin-1a	g3757.t1	5.00E-02
	Cadherin-5	g21848.t1	7.36E-03
	BMP-binding endothelial regulator protein	g7031.t1	2.71E-02
	Bone morphogenetic protein 4	g189.t1	5.73E-03
	Bone morphogenetic protein 5	g11279.t1	2.76E-03
	Bone morphogenetic protein 8A	g14806.t1	9.26E-03
	Bone morphogenetic protein receptor type-1A	g14843.t1	9.45E-05
	Transforming growth factor beta-2	g2430.t1	8.22E-03
	Transforming growth factor beta-3	g14668.t1	2.68E-02
	Rho-related GTP-binding protein RhoB	g5763.t1	1.23E-09
	Thrombospondin-1	g5480.t1	3.33E-02
	Mothers against decapentaplegic homolog 6	g4737.t1	6.93E-13
	Mothers against decapentaplegic homolog 6	g4739.t1	3.88E-11
	Mothers against decapentaplegic homolog 3	g3356.t1	4.28E-03

Of the five groups of DEGs, two groups were highly expressed in the gill: six genes for acid-base balance, and 11 genes for ion homeostasis (Table 3), reflecting the critical role that the gill plays in acid-base and ion regulation. Three groups of genes were highly expressed in the air-breathing organ: eight “elastic fiber formation” genes, eight hemoglobin genes, and 18 genes involved in angiogenesis (Table 3). The eight hemoglobin genes (four alpha subunit genes and four beta subunit genes) were found to be dramatically up-regulated in the air-breathing organ, as compared to in the gill which is primarily an aquatic respiratory organ (Additional file 1: Table S12), demonstrating that the air-breathing organ is highly committed to the respiratory processes for oxygen transport.

## Discussion

In this study, we sequenced and assembled the genome sequence from walking catfish, which provided a comprehensive understanding of this species at the genomic and evolutionary levels. Comparative analysis with 11 non-air-breathing fish species suggested its adaptive evolution in terms of gene expression and nitrogenous waste metabolic processes. It has been well documented that different organisms can achieve diverse and specific responses to multiple environmental stresses by regulating gene expression to maintain homeostasis [37–39]. In addition, ammonia is the main nitrogenous waste in fishes, which is highly toxic and needs to be excreted promptly or converted to other less-toxic chemicals. *C. batrachus* usually inhabits water bodies with high levels of ammonia and sometimes dwells inside mudflats or “walks” on the land, during which excretion of ammonia directly into the aqueous environment through the gill is impossible [7]. To adapt to the hyper-ammonia stress, *C. batrachus* is highly tolerant to external ammonia and can convert ammonia into non-essential amino acids and less-toxic urea through the ornithine-urea cycle (OUC) [7, 40–42]. Interestingly, the gene argininosuccinate synthase (*ass*) encoding one of the key enzymes in the OUC was found to be under positive selection in comparison with non-air-breathing fish species, implying the necessity of this adaptive strategy for air-breathing walking catfish for survival in hyper-ammonia environments. Further, the wide use of urea as the main nitrogenous waste product in amphibians, some turtles and mammals has been hypothesized to be a key evolutionary process for transition from water to land [43, 44], suggesting the importance of urea excretion among the adaptations of walking catfish to the terrestrial life.

Although no specific genes that are present only in the air-breathing walking catfish were found, three important genes (*mb*, *ora1* and *sult6b1*) were found to be significantly expanded in the genome - with 15, 15, and 12 copies, respectively - compared to non-air-breathing fishes that possess only 1–2 copies of these genes. We believe that the noted gene expansions are real, not caused by inaccuracies of genome assembly. In spite of being a draft genome sequence, the genome assembly is of high quality. The same assembly results, especially in the regions containing the noted gene expansions, were achieved by using different de novo genome assemblers (ABySS and ALLPATHS-LG), suggesting the accuracy of the assembly, and therefore the accuracy for assessment of tandem duplications. The genome sequencing was conducted using DNA template from a single individual, and thus two allelic variations of sequences are expected. Multiple sequence alignments (Additional file 1: Figure S1, Additional file 1: Figure S3) and phylogenetic trees (Fig. 3, Additional file 1: Figure S2) indicated that the amino acid sequences of the duplicated genes are divergent. Additionally, duplicates of *mb* and *sult6b1* are located on different scaffolds with different flanking genes, all of which also have transcriptomic evidence (Table 2).

Gene expansion may be a “handy” approach for genome evolution to rapidly adapt to environments, especially stressful conditions. Tandem gene duplication usually results from unequal crossing over, but the fixation of duplicated genes is a time-consuming process, which is determined by the functions of the gene duplicates [45, 46]. If the functions are beneficial and essential, strong purifying selection would prevent duplicated genes from pseudogenization or neofunctionalization during a long-term evolutionary process, resulting in very similar sequences among those duplicates [45]. As predicted by Susumo Ohno (1970) [47], our study provides support for the view that tandem duplications provide important evolutionary mechanisms for adaptation and diversification [48]. It appeared that the “sand-bagging” style of gene expansion could be a possible mechanism for evolution of aquatic genomes to cope with stressful environments, especially those that pose life-or-death consequences. In a recent study, Xu et al. [28] reported drastic expansion of egg-coat proteins and natriuretic peptide receptors in Amur ide *Leuciscus waleckii* that lives under extremely alkaline conditions.

The expansion of myoglobin genes in *C. batrachus* may be consistent with its frequent exposure to low-oxygen habitats and occasional terrestrial migration. Myoglobin, as an oxygen binding protein predominantly in skeletal and cardiac muscles, is able to bind and store oxygen and facilitate the delivery of oxygen to peripheral tissues [49, 50]. Millikan [51] reported that myoglobin maintains balance in periods of fluctuating oxygen supply and demand through rapid oxygenation and deoxygenation. Additionally, myoglobin maintains a steady level of oxygenation to the mitochondria during muscle contraction [49, 52]. Although the copy number for myoglobin is not expanded in mammals, many studies indicate that it is highly regulated, with higher levels of expression in the skeletal muscle of hypoxia-tolerant animals such as deep-diving and high-elevation mammals compared to surface and lowland relatives [53, 54]. In fish species, comparative studies have been conducted between *mb*-high sea raven (*Hemitripterus americanus*) and *mb*-low ocean pout (*Macrozoarces americanus*) [55] and between *mb*-present icefsh (*Chionodraco rastrospinosus*) and *mb*-absent icefish (*Chaenocephalus aceratus*) [56]. Both studies indicated that myoglobin plays a critical role in maintaining oxygen consumption in the heart and enhances cardiac performance. In addition to those functions of oxygen storage and transport, myoglobin also was found to be involved in protecting mitochondrial respiration from nitric-oxide (NO) inhibition [57] and in scavenging of reactive oxygen species (ROS) [58]. Especially during hypoxia and subsequent re-oxygenation periods, the production of ROS increased significantly [59, 60]. One prominent example was found in common carp (*Cyprinus carpio*), in which the additional myoglobin isoform *mb2* played a protective role against ROS in the brain [32, 61]. Similarly in West African lungfish, notable myoglobin expression in the brain was observed, and the cell-level experiments also suggested a key role of myoglobin in protecting the tissues from ROS [35].

Olfaction is an important sense for fish to recognize odorants due to the great ability of water to carry chemical compounds, and their reduced visual ability in turbid environments. Some fishes, including catfishes, have chemoreceptors on their barbels and anterior surfaces of the body. Unlike mammals possessing a main olfactory epithelium (MOE) and a vomeronasal organ (VNO) to express different types of chemoreceptors, fish only have MOE [62–65]. The separation of MOE and VNO in terrestrial vertebrates may have resulted evolutionarily from the segregation of distinct classes of neurons that were differentially positioned in the MOE of aquatic vertebrates [65]. Furthermore, the *ora* genes in fish species are homologs of the vomeronasal receptor 1 (*v1r*) in mammals [66]. Surprisingly, the *ora* genes are very conserved in fish species, with very rare gene duplication events [36], while mammalian genomes harbor hundreds of *v1r* genes [67]. In most cases, fish species possess six *ora* genes with *ora1*-*ora2*, *ora3*-*ora4* and *ora5*-*ora6* forming three phylogenetic clades (Fig. 3b, Additional file 1: Figure S2), suggesting a close evolutionary relationship within each gene pair [36]. In the *C. batrachus* genome, we identified all six *ora* genes, but *ora1* was expanded with fifteen tandem copies. Interestingly, the expansions of *v1r* in mammals also were clustered as tandem duplications [68] and fell within the *ora1*-*ora2* gene-pair clade of teleost species (Fig. 3b, Additional file 1: Figure S2), suggesting that the gaining of *ora3*-*ora6* genes in aquatic species might be due to the aquatic lifestyle [36]. Also, coelacanth (*Latimeria chalumnae*), an ancient lobe-finned fish that is thought to be evolutionarily close to tetrapods, not only possesses all the *ora* genes, but also experienced an expansion in the *ora1*-*ora2* gene-pair clade [69, 70] (Fig. 3b, Additional file 1: Figure S2), which is similar to *C. batrachus*. These related observations may suggest that the expansion of *ora1* genes in *C. batrachus* might be associated with adaptation for transition from water to land, allowing recognition of airborne chemicals to help better detect threats and locate water sources.

Sulfotransferase 6b1 encodes a key enzyme for the process of detoxifying and eliminating xenobiotics. Aquatic habitats are increasingly polluted world-wide, and such contaminants adversely affect the health of aquatic animals [71]. *C. batrachus*, as an air-breathing fish, not only suffers from the same toxins in the water as other aquatic animals, but also endures higher concentrations of toxic chemicals in drying water bodies as well as from the land. To counteract the toxic effects of these xenobiotics, complex enzyme-based mechanisms are needed to detoxify and eliminate these chemical compounds. Sulfotransferases function by conjugation of a sulfate group on the target xenobiotics to increase their hydrophilicity for excretion [72]. *C. batrachus* demonstrated an overall higher tolerance to three widely distributed xenobiotics than two other air-breathing fish species, the Asian stinging catfish (*Heteropneustes fossilis*) and spotted snakehead (*Channa punctatus*) [73]. Rainbow trout (*Oncorhynchus mykiss*) fry and Japanese flounder (*Paralichthys olivaceus*) showed significantly increased expression of *sult6b1* after exposure to diesel and the water-accommodated fraction of crude oil, respectively, indicating that *sult6b1* does function in eliminating toxic chemicals in fish species [74, 75]. Taken together, the expansion of *sult6b1* may play crucial roles in protecting *C. batrachus* from the deleterious effects of different xenobiotics from the aquatic and terrestrial environments.

It has been suggested that air breathing has evolved as an adaptation for fish to cope with hypoxic conditions, and consequently it provided an essential first step to terrestrial habitation in the evolution of vertebrates [76–80]. Consequently, we sequenced and compared the transcriptomes of the gill and the air-breathing organ to investigate the mechanism of aerial respiration. It showed that acid-base balance and ion homeostasis related genes were up-regulated in the gill, while elastic fiber formation, oxygen binding and transport, and angiogenesis genes were up-regulated in the air-breathing organ. Acid-base regulation in vertebrates is coupled to carbon dioxide (CO_2_) excretion through the reversible hydration/dehydration reactions of CO_2_ and the acid-base equivalents H^+^ and HCO_3_^−^ by carbonic anhydrase (CA) (Table 3). It is always linked to ion regulation because acid-base compensation depends on the transfer of H^+^ and HCO_3_^−^ in exchange for Na^+^ and Cl^−^ across the gill, respectively [81–83]. Both acid-base balance and ion homeostasis contribute greatly to maintaining the well-balanced conditions for efficient aerial respiration by the air-breathing organ.

Elastic fibers are important structural components of the arborescent organ [84, 85], while hemoglobin genes and genes involved in angiogenesis apparently provide a functional basis for *C. batrachus* to cope with low oxygen in the terrestrial environment. The hemoglobin genes were expressed dramatically higher in the air-breathing organ than in the gill of walking catfish (Additional file 1: Table S12), suggesting their important roles in sufficient oxygen supply during air-breathing activities. Additionally, 18 genes involved in angiogenesis were found differentially expressed in the air-breathing organ (Table 3). It is also well documented that angiogenesis plays a critical role in respiratory function for accessory air-breathing organs of fishes [76, 86, 87]. Also, the air-breathing organ of *C. batrachus* is highly vascularized on the surface, and the capillaries extensively bulge out onto the surface to facilitate gas exchange between blood and atmospheric air [10, 88]. Consequently, heightened angiogenesis may be one additional adaptation for the air-breathing organ to maintain high efficiency of air exchange. Overall, it appears that the strategy during adaptive evolution of *C. batrachus* to the transition from aquatic to terrestrial environment may be through the coupling of high expression of hemoglobin and angiogenesis genes for oxygen transport with expansion of myoglobin genes for oxygen uptake and storage in the peripheral tissues.

## Conclusions

The walking catfish is an aquatic species but can move about on land without a lung. As such, it is a remarkable model to investigate the transition from the aquatic to the terrestrial environment, and the adaptation to terrestrial life. Through whole-genome sequencing analysis, we did not find any specific genes that were present in this air-breathing fish, but absent in non-air-breathing fishes. However, highly suggestive gene family expansions (mostly in tandem) were found within the *C. batrachus* genome. Of particular interest is the expansion of the oxygen-storage protein myoglobin gene, with 15 copies, while non-air-breathing fishes have only one to two copies of this gene. West African lungfish was found to harbor seven copies of the myoglobin gene, and this expansion of myoglobin genes was believed to be crucially important for its adaptation to survive hypoxic periods [35]. Therefore, it is likely that the expansion of myoglobin genes may be a possible mechanism for the water-to-land transition. Additionally, the olfactory receptor related to class A G protein-coupled receptor 1 and the sulfotransferase 6b1 genes were found to be highly expanded, with the former being related to the olfactory sense and the latter to provide resistance to xenobiotics.

The coupling of enhanced oxygen transport, and oxygen uptake and storage may be important for the water-to-land transition. Hemoglobin genes were found to be expressed at much higher levels in the air-breathing organ of *C. batrachus* than in its gill. While the hemoglobin genes are also highly duplicated, the number of paralogous copies in the air-breathing *C. batrachus* is not larger than that in the non-air-breathing fishes. Instead, regulation appeared to be at the transcriptional level, where hemoglobin RNAs were transcribed many times more highly in the air-breathing organ, ensuring the greater capacity for oxygen transport. In addition, many genes involved in angiogenesis were found to be expressed at much higher levels in the air-breathing organ than in the gill of *C. batrachus*, providing the structural basis for expanded blood vessel systems for gas exchange. Taken together, the evolution for the water-to-land transition seemed to involve mostly expanded oxygen storage genes through gene duplications and transcriptional up-regulation of oxygen transport genes.

## Methods

### *C. batrachus* samples and genome sequencing

One wild walking catfish (*Clarias batrachus*) was collected from Florida, USA in June of 2014, and the fish was euthanized with MS-222 (200 mg/l) before blood sampling. Genomic DNA was extracted from blood cells using the DNeasy Blood and Tissue kit (Qiagen, CA). One short-insert (180 bp) paired-end library and one long-insert (3 kb) library were constructed. Each library was subjected to one lane of 2 × 100 bp read-length run on an Illumina HiSeq 2500 sequencer at HudsonAlpha (Huntsville, AL, USA).

### Genome assembly and assessment

After raw reads were evaluated in FastQC v0.11.4 [89], low-quality bases and adapter sequences were trimmed from the raw sequences using cutadapt v1.8.1 [90], and then reads with length shorter than 30 bases after trimming were removed. The genome sequence was assembled by ABySS v1.5.2 [91] with *k*-mers ranging from 40 to 70 in size and ALLPATHS-LG [92]. Finally, *k*-mer size of 61 yielded the best assembly results using ABySS. To increase scaffold length, we selected assembled sequences with longer contig N50 from ALLPATHS-LG for scaffolding by SSPACE v3.0 [93]. Finally, the paired-end reads were utilized to fill the gaps in the scaffolds with Gapfiller v1.10 [94]. Genome size was estimated in the ALLPATHS-LG using trimmed paired-end reads.

To assess the quality of the assembly results, CEGMA v2.5 (Core Eukaryotic Genes Mapping Approach) [19] was employed to evaluate the completeness of the *C. batrachus* draft genome sequence. In other words, 248 highly conserved core eukaryotic genes (CEGs) from six genomes of model systems (*Homo sapiens*, *Drosophila melanogaster*, *Caenorhabditis elegans*, *Arabidopsis thaliana*, *Saccharomyces cerevisiae* and *Schizosaccharomyces pombe*) [19] were mapped to the genome assembly to display the percentage of the CEGs present in the *C. batrachus* genome. Another assessment procedure, BUSCO v1.22 (Benchmarking Universal Single-Copy Orthologs) [20], was used to evaluate the completeness of genome assembly by 3023 genes selected from orthologous groups with single-copy orthologs in > 90% of available vertebrate genomes. Then, the five longest scaffolds of assembly resulting from another assembler (ABySS) were mapped against the genome sequence using NUCmer in MUMmer v3.23 [95] to evaluate the aligned identity.

### Genome annotation

A de novo repeat library was constructed using RepeatModeler v1.0.8 (http://www.repeatmasker.org/RepeatModeler.html), which contains two de novo repeats-finding programs, RECON [96] and RepeatScout [97]. Next, RepeatMasker v4.0.6 (http://www.repeatmasker.org/) was used to predict and categorize repeat sequences in the *C. batrachus* genome with the repeat library constructed from RepeatModeler. The Jukes-Cantor model [98] was used to estimate the average number of substitutions per site for each fragment based on the divergence levels from the results of RepeatMasker. For the subsequent genome annotation, the genome sequence was masked with “N” in the repeat regions except for low-complexity DNA or simple repeats.

AUGUSTUS v3.2.1 [99] was used for the ab initio predictions of genes on the repeat-masked genome. Gene model parameter sets for AUGUSTUS were trained from genes in zebrafish (*Danio rerio*). The predicted genes with length less than 30 amino acids were removed. The remaining predicted amino acid sequences were aligned to entries in the NCBI non-redundant (nr) protein database and SwissProt and TrEMBL subsets of the UniProt database [25] by BLASTP with an *E*-value cut-off of 1 × 10^− 5^ to identify homologous genes. Functional categories of GO terms were determined by Blast2GO version 4.0.7 [100], and the KEGG Automatic Annotation Server (KAAS) (http://www.genome.jp/tools/kaas/) BBH (bi-directional best hit) method [101] was used to perform a biological pathway analysis.

### Comparative genomic analysis

The protein sequences of channel catfish (*Ictalurus punctatus*; NCBI version IpCoco_1.2) [23] were downloaded for comparison with those of *C. batrachus* to determine *C. batrachus*-specific genes in the catfish lineage. The methodology was based on the one used in the channel catfish genome paper [23]. First, the proteins from both catfishes were sent to OrthoFinder v1.0.2 [102] for an all-to-all BLASTP comparison with an *E* -value threshold of 1 × 10^− 5^ and subsequent clustering into orthogroups based on the MCL algorithm. Next, a further round of BLASTP searches was performed using the genes not included in the orthogroups to query against the genes in the orthogroups within the same species with an *E* -value threshold of 1 × 10^− 10^. In the end, reciprocal BLASTP searches between them with an *E* -value threshold of 1 × 10^− 5^ were performed using genes with no hits from last step as queries. The remaining genes in *C. batrachus* were considered as species-specific genes and kept for a further GO-term overrepresentation test using PANTHER version 11 [103] with the best homologous gene ID from zebrafish.

Protein sequences of an additional 10 teleost fish species including zebrafish (*Danio rerio*; Ensembl version GRCz10), three-spined stickleback (*Gasterosteus aculeatus*; Ensembl version BROAD S1), green spotted pufferfish (*Tetraodon nigroviridis*; Ensembl version TETRAODON8.0), Japanese pufferfish (*Takifugu rubripes*; Ensembl version FUGU4.0), medaka (*Oryzias latipes*; Ensembl version HdrR), Atlantic cod (*Gadus morhua*; Ensembl version fadMor1), Mexican cave fish (*Astyanax mexicanus*; Ensembl version AstMex102), Nile tilapia (*Oreochromis niloticus*; Ensembl version Orenil1.0), southern platyfish (*Xiphophorus maculatus*; Ensembl version Xipmac4.4.2) and amazon molly (*Poecilia formosa*; Ensembl version Poecilia_formosa-5.1.2) were downloaded for inferring orthologues. The longest protein sequence was selected for each gene among the eleven sequenced fish species (channel catfish included). After combination with *C. batrachus* protein sequences, all the sequences were sent to OrthoFinder v1.0.2 [102] to identify orthologues and orthogroups among these species. Genes that are present in the *C. batrachus* genome but absent from the non-air-breathing fishes were obtained. Next, these genes specific to *C. batrachus* were searched with all existing sequences from non-air-breathing fishes in the NCBI database to find the genes that are present only in the *C. batrachus* genome.

Single-copy genes were extracted from all the species to construct a phylogenetic tree. Multiple sequence alignments were performed using MUSCLE v3.8.31 [104] for protein alignments and PAL2NAL [105] for codon alignments. We used Gblock v0.91b [106] to eliminate poorly aligned positions and divergent regions of the alignments. Final alignments with length shorter than 50 amino acids for protein alignments and 150 bp for codon alignments were removed. AMAS [107] was performed to combine all the refined alignments into a concatenated alignment. PartitionFinder v2.0.0 was used to determine the best substitution model for each gene with the parameter of -rcluster-percent = 20.0 [108]. Then we used the rapid bootstrap algorithm with a thorough ML search (−f a) and 100 bootstrap replicates in RAxML v8.2.9 [109] to construct a maximum likelihood tree for those single-copy genes.

To determine positively-selected genes in *C. batrachus*, the single-copy genes were collected for analyzing the d_N_/d_S_ ratio. The values of d_N_, d_S_ and d_N_/d_S_ ratio were estimated using the codeml program in the PAML package version 4.9 [110]. Sequence alignments with d_S_ value greater than 2 were removed to avoid distortion of the d_N_/d_S_ ratio by saturation of synonymous substitutions [111]. The values of d_N_/d_S_ between each species branch and the ancestral branch from 150 randomly picked genes were estimated with 10,000 bootstrap replicates to evaluate the magnitude of natural selection acting on each species. Then, a branch-site model [112] was used to designate *C. batrachus* as a “foreground” branch and the rest of the species as “background”. A likelihood ratio test (LRT) was computed to compare a model that allows sites to be under positive selection (ω > 1) on the foreground branch with the null model that allows sites to be under negative selection (ω < 1) and to evolve neutrally (ω = 1) with a posterior probability greater than 0.95 based on Bayes Empirical Bayes (BEB) results [113]. After an FDR multiple-testing correction, the positively selected genes (FDR < 0.05) were selected for further GO-term enrichment analysis by Blast2GO version 4.0.7 [100] with the whole reference gene set as the background for statistical analysis. After annotating *C. batrachus* genes with the best homologous zebrafish genes by BLASTP and Ensembl BioMart [114], the Reactome pathway database v60 [115] was used for further pathway enrichment analysis.

### Gene family analysis

Orthologous genes were sent to the CAFÉ v3.0 [116] program to assess gene family expansion and contraction (−r 1000 -s). A family-wide *P*-value of less than 0.01 and a branch-specific *P*-value of less than 0.001 was utilized to identify gene family expansion in the *C. batrachus* genome. The expanded families in the *C. batrachus* genome were searched against the NCBI database to exclude false-positive expansions due to the limited number of species in previous analyses, during which the numbers of genes in tetraploid species were divided by two for direct comparisons.

For those significantly expanded genes, phylogenetic trees were constructed to display the gene expansions. The accession numbers of all the protein sequences used in the phylogenetic analyses are listed in Additional file 1: Table S15. Multiple sequence alignments were performed using ClustalW [117] in MEGA6 [118], and ProtTest v3.4 [119] was utilized to select the best model for constructing phylogenetic trees (Additional file 1: Table S15). Phylogenetic analysis was conducted using MEGA6 with the maximum likelihood method. Bootstrapping with 1000 replications was conducted to evaluate the phylogenetic tree. RNA-Seq datasets from gill and the air-breathing organ in this study and also from brain (SRR2057993), head kidney (SRR2057995) and liver (SRR2052655) in the public database were mapped to the genome sequence to estimate the Fragments Per Kilobase per Million mapped fragments (FPKM) metric for those expanded genes respectively by TopHat 2.0.10 and Cufflinks 2.1.1 [120–122].

### Comparative transcriptomic analysis between the gill and the air-breathing organ

Wild *C. batrachus* individuals (70–136 g) were collected from Miami, Florida, USA in October of 2015. Tissue samples were collected after euthanasia using MS-222. The tissue samples from gill and air-breathing organ were kept in the RNAlater solution (Ambion) to prevent RNA degradation. Total RNAs were extracted from tissues of five individuals using the RNeasy Plus Universal Mini kit (Qiagen, CA) according to manufacturer’s instructions, and then the RNAs from five samples were mixed in equal amounts for RNA-Seq at HudsonAlpha (Huntsville, AL, USA). Standard Poly-A libraries were prepared, and 125 bp paired-end reads were generated using Illumina HiSeq 2500 sequencing platform. Raw reads were filtered with the parameters of base quality ≥20 and trimmed length ≥ 36 bp by Trimmomatic v0.32 [123]. All the trimmed reads from both tissues were mapped to 22,914 coding sequences predicted from genome assembly by the CLC Genomics Workbench software package [124]. The parameters for mapping were set as 90% or greater sequence identity with a maximum of two mismatches. The number of total mapped reads on each contig and reads per kilobase per million mapped reads (RPKM) were collected. After normalization of RPKM values, fold-changes were estimated to exhibit differentially expressed patterns of gene expression between the air-breathing organ and gill transcriptomes with a *P*-value < 0.05 using proportions-based Kal’s test [125] in the CLC Genomics Workbench software package. Transcripts with fold-change values greater than 2 were regarded as differentially expressed genes for subsequent analysis. Blast2GO version 4.0.7 [100] was used with default settings for the over-representation analysis of GO terms among the differentially expressed genes in the air-breathing organ and gill, and Reactome pathway database v60 [115] was used for further pathway enrichment analysis to indicate the functional differences between air-breathing organ and gill.

### Real-time PCR validation of differentially expressed genes

To confirm the accuracy of the RNA-Seq analysis, quantitative real-time PCR (qRT-PCR) analysis was conducted. Total RNAs were extracted from the gill and air-breathing organ using the RNeasy Plus Universal Mini kit (Qiagen, CA) following the manufacturer’s instructions. After quantification with a Nanodrop spectrophotometer (Thermo Scientific), cDNA was synthesized with a final concentration of 50 ng/μL using the iScript cDNA Synthesis Kit (Quanta BioSciences) based on the manufacturer’s protocol. The primers used in qRT-PCR are listed in Additional file 1: Table S16. Amplification was performed on a CFX96 real-time PCR Detection System (Bio-Rad, CA). The thermal cycling profile consisted of an initial denaturation at 95 °C for 30 s, 40 cycles of denaturation at 94 °C for 5 s and an appropriate annealing/extension temperature at 60 °C for 10 s, and 72 °C for 5 s, followed by dissociation curve analysis to validate the specificity of amplified products. The 28S ribosomal RNA (rRNA) [126] (accession number JK488212) was used as the reference gene. Relative fold-changes for each gene were calculated in the Relative Expression Software Tool (REST) version 2009 [127] based on the values of cycle threshold (C_t_) from real-time PCR.

## Additional file

Additional file 1: **Figure S1**. Multiple sequence alignment of myoglobin genes in the genomes of *Clarias batrachus*, *Danio rerio*, *Ictalurus punctatus* and *Astyanax mexicanus*. **Figure S2**. Detailed phylogenetic tree of olfactory receptor related to class A G protein-coupled receptor (*ora*) with species names and sequence names for Figure 3b. **Figure S3**. Multiple sequence alignment of olfactory receptor related to class A G protein-coupled receptor 1 (*ora1*) in the genomes of *Clarias batrachus*, *Danio rerio*, *Ictalurus punctatus* and *Astyanax mexicanus*. **Figure S4**. Syntenic analysis of myoglobin gene (*mb*) using genomic information of *Danio rerio*, *Ictalurus punctatus* and *Clarias batrachus*. **Figure S5**. Comparison of relative fold changes between air-breathing organ and gill in *Clarias batrachus* after normalization to 28S rRNA using RNA-Seq datasets and qRT-PCR. **Table S1**. Completeness of genome assembly assessed by CEGMA and BUSCO. **Table S2**. Mapping of five longest ABySS scaffolds to genome assembly. **Table S3**. Repetitive elements in the *Clarias batrachus* genome. Table S4. Orthogroups shared between species by OrthoFinder. **Table S5**. Specific genes in the *Clarias batrachus* genome compared with that of channel catfish (*Ictalurus punctatus*). **Table S6**. GO terms significantly enriched in the specific genes in the *Clarias batrachus* genome compared with that of channel catfish (*Ictalurus punctatus*). **Table S7**. Positively selected genes in the *Clarias batrachus* genome compared with those of 11 non-air-breathing teleost fish. **Table S8**. GO terms significantly enriched in the positively selected genes in the *Clarias batrachus* genome. **Table S9**. Expanded genes in the *Clarias batrachus* genome. **Table S10**. Summary of transcriptome sequencing data. **Table S11**. Expression values (RPKM) of all the genes in the transcriptomes of the gill and the air-breathing organ. **Table S12**. Differentially expressed genes in the transcriptome of the air-breathing organ compared with that of the gill. **Table S13**. GO terms significantly enriched in the differentially expressed genes comparing the transcriptome of the air-breathing organ with that of the gill. **Table S14**. Pathways significantly enriched in the differentially expressed genes comparing the transcriptome of the air-breathing organs with that of the gill. **Table S15**. Accession numbers of protein sequences and models used in the construction of phylogenetic trees of expanded genes. **Table S16**. Primer sequences used for qRT-PCR. (ZIP 5238 kb)

## Abbreviations

BUSCO: Benchmarking Universal Single-Copy Orthologs
CEG: Core Eukaryotic Gene
DEG: Differentially Expressed Gene
FPKM: Fragments Per Kilobase per Million mapped fragments
GO: Gene Ontology
LINE: Long Interspersed Elements
LRT: Likelihood Ratio Test
LTR: Long Terminal Repeats
MOE: Main Olfactory Epithelium
NR: Non-Redundant
OUC: Ornithine Urea Cycle
qRT-PCR: Quantitative Real-Time PCR
ROS: Reactive Oxygen Species
RPKM: Reads Per Kilobase per Million mapped reads
rRNA: Ribosomal RNA
SINE: Short Interspersed Elements
VNO: Vomeronasal Organ

## References

[1] Srivastava S, Kushwaha B, Prakash J, Kumar R, Nagpure N, Agarwal S, Pandey M, Das P, Joshi C, Jena J (2016). Development and characterization of genic SSR markers from low depth genome sequence of *Clarias batrachus* (Magur). J Genet.

[2] Dahanukar N, Raut R, Bhat A (2004). Distribution, endemism and threat status of freshwater fishes in the Western Ghats of India. J Biogeogr.

[3] Islam MN, Islam MS, Alam MS (2007). Genetic structure of different populations of walking catfish (*Clarias batrachus* L.) in Bangladesh. Biochem Genet.

[4] Khedkar GD, Reddy ACS, Mann P, Ravinder K, Muzumdar K (2010). *Clarias batrachus* (Linn. 1758) population is lacking genetic diversity in India. Mol Biol Rep.

[5] Allen DJ. *Clarias batrachus*. The IUCN Red List of Threatened Species 2011: e.T166613A6247551. (http://dx.doi.org/10.2305/IUCN.UK.2011-1.RLTS.T166613A6247551.en). Accessed 17 Dec 2016.

[6] Courtenay W Jr, Hensley D, Taylor J, McCann J. Distribution of exotic fishes in North America. Wiley; 1986. p. 675–698.

[7] Saha N, Ratha B (2007). Functional ureogenesis and adaptation to ammonia metabolism in Indian freshwater air-breathing catfishes. Fish Physiol Biochem.

[8] Das B (1928). The bionomics of certain air-breathing fishes of India, together with an account of the development of their air-breathing organs. Phil Trans R Soc Lond B.

[9] Courtenay WR, Sahlman HF, Miley WW, Herrema DJ (1974). Exotic fishes in fresh and brackish waters of Florida. Biol Conserv.

[10] Munshi J (1961). The accessory respiratory organs of *Clarias batrachus* (Linn.). J Morphol.

[11] Lewis S (1979). The morphology of the accessory air-breathing organs of the catfish, *Clarias batrachus*: a SEM study. J Fish Biol.

[12] Saha N, Ratha B (1998). Ureogenesis in Indian air-breathing teleosts: adaptation to environmental constraints. Comp Biochem Physiol A Mol Integr Physiol.

[13] Kelley JL, Yee M-C, Brown AP, Richardson RR, Tatarenkov A, Lee CC, Harkins TT, Bustamante CD, Earley RL (2016). The genome of the self-fertilizing mangrove rivulus fish, *Kryptolebias marmoratus*: a model for studying phenotypic plasticity and adaptations to extreme environments. Genome Biol Evol..

[14] You X, Bian C, Zan Q, Xu X, Liu X, Chen J, Wang J, Qiu Y, Li W, Zhang X (2014). Mudskipper genomes provide insights into the terrestrial adaptation of amphibious fishes. Nat Commun.

[15] Randall D, Ip Y, Chew S, Wilson J (2004). Air breathing and ammonia excretion in the giant mudskipper, *Periophthalmodon schlosseri*. Physiol Biochem Zool.

[16] Wright PA (2012). Environmental physiology of the mangrove rivulus, *Kryptolebias marmoratus*, a cutaneously breathing fish that survives for weeks out of water. Integr Comp Biol.

[17] Jianxun C, Xiuhai R, Qixing Y (1991). Nuclear DNA content variation in fishes. Cytologia.

[18] Hinegardner R, Rosen DE. Cellular DNA content and the evolution of teleostean fishes. Am Nat. 1972:621–44.

[19] Parra G, Bradnam K, Korf I (2007). CEGMA: a pipeline to accurately annotate core genes in eukaryotic genomes. Bioinformatics.

[20] Simão FA, Waterhouse RM, Ioannidis P, Kriventseva EV, Zdobnov EM (2015). BUSCO: assessing genome assembly and annotation completeness with single-copy orthologs. Bioinformatics.

[21] Vinogradov AE (1998). Genome size and GC-percent in vertebrates as determined by flow cytometry: the triangular relationship. Cytometry.

[22] Tarallo A, Angelini C, Sanges R, Yagi M, Agnisola C, D’Onofrio G (2016). On the genome base composition of teleosts: the effect of environment and lifestyle. BMC Genomics.

[23] Liu Z, Liu S, Yao J, Bao L, Zhang J, Li Y, Jiang C, Sun L, Wang R, Zhang Y (2016). The channel catfish genome sequence provides insights into the evolution of scale formation in teleosts. Nat Commun.

[24] Li WH, Graur D. Fundamentals of molecular evolution. 1st ed. Sinauer Associates; 1991.

[25] Boeckmann B, Bairoch A, Apweiler R, Blatter M-C, Estreicher A, Gasteiger E, Martin MJ, Michoud K, O'Donovan C, Phan I (2003). The SWISS-PROT protein knowledgebase and its supplement TrEMBL in 2003. Nucleic Acids Res.

[26] Toksoz D, Merdek K (2001). The rho small GTPase: functions in health and disease. Histol Histopathol.

[27] Narumiya S (1996). The small GTPase rho: cellular functions and signal transduction. J Biochem.

[28] Xu J, Li J-T, Jiang Y, Peng W, Yao Z, Chen B, Jiang L, Feng J, Ji P, Liu G. Genomic basis of adaptive evolution: the survival of Amur ide (*Leuciscus waleckii*) in an extremely alkaline environment. Mol Biol Evol. 2016;34(1):145–59. 10.1093/molbev/msw230 PMC5854124 28007977

[29] Fuchs C, Burmester T, Hankeln T (2006). The amphibian globin gene repertoire as revealed by the *Xenopus* genome. Cytogenet Genome Res.

[30] Sidell BD, O'Brien KM (2006). When bad things happen to good fish: the loss of hemoglobin and myoglobin expression in Antarctic icefishes. J Exp Biol.

[31] Hoffmann FG, Opazo JC, Storz JF (2011). Differential loss and retention of cytoglobin, myoglobin, and globin-E during the radiation of vertebrates. Genome Biol Evol.

[32] Fraser J, de Mello LV, Ward D, Rees HH, Williams DR, Fang Y, Brass A, Gracey AY, Cossins AR (2006). Hypoxia-inducible myoglobin expression in nonmuscle tissues. Proc Natl Acad Sci U S A.

[33] Roesner A, Mitz SA, Hankeln T, Burmester T (2008). Globins and hypoxia adaptation in the goldfish, *Carassius auratus*. FEBS J.

[34] Schwarze K, Campbell KL, Hankeln T, Storz JF, Hoffmann FG, Burmester T (2014). The globin gene repertoire of lampreys: convergent evolution of hemoglobin and myoglobin in jawed and jawless vertebrates. Mol Biol Evol.

[35] Koch J, Lüdemann J, Spies R, Last M, Amemiya CT, Burmester T (2016). Unusual diversity of myoglobin genes in the lungfish. Mol Biol Evol.

[36] Saraiva LR, Korsching SI (2007). A novel olfactory receptor gene family in teleost fish. Genome Res.

[37] Murray JI, Whitfield ML, Trinklein ND, Myers RM, Brown PO, Botstein D (2004). Diverse and specific gene expression responses to stresses in cultured human cells. Mol Biol Cell.

[38] De Nadal E, Ammerer G, Posas F (2011). Controlling gene expression in response to stress. Nature Rev Genet.

[39] Atkinson B. Changes in eukaryotic gene expression in response to environmental stress. Academic Press; 2012.

[40] Saha N, Dutta S, Haussinger D (2000). Changes in free amino acid synthesis in the perfused liver of an air-breathing walking catfish, *Clarias batrachus* infused with ammonium chloride: a strategy to adapt under hyperammonia stress. J Exp Zool.

[41] Saha N, Das L (1999). Stimulation of ureogenesis in the perfused liver of an Indian air-breathing catfish, *Clarias batrachus*, infused with different concentrations of ammonium chloride. Fish Physiol Biochem.

[42] Saha N, Dutta S, Bhattacharjee A (2002). Role of amino acid metabolism in an air-breathing catfish, *Clarias batrachus* in response to exposure to a high concentration of exogenous ammonia. Comp Biochem Physiol B Biochem Mol Biol.

[43] Wright PA (1995). Nitrogen excretion: three end products, many physiological roles. J Exp Biol.

[44] Amemiya CT, Alfoldi J, Lee AP, Fan S, Philippe H, Maccallum I, Braasch I, Manousaki T, Schneider I, Rohner N (2013). The African coelacanth genome provides insights into tetrapod evolution. Nature.

[45] Zhang J (2003). Evolution by gene duplication: an update. Trends Ecol Evol.

[46] Kimura M (1983). The neutral theory of molecular evolution.

[47] Ohno S (1970). Evolution by gene duplication.

[48] Meyer A, Schartl M (1999). Gene and genome duplications in vertebrates: the one-to-four (−to-eight in fish) rule and the evolution of novel gene functions. Curr Opin Cell Biol.

[49] Millikan G (1939). Muscle hemoglobin. Physiol Rev.

[50] Wittenberg JB (1970). Myoglobin-facilitated oxygen diffusion: role of myoglobin in oxygen entry into muscle. Physiol Rev.

[51] Millikan G. Experiments on muscle haemoglobin *in vivo*; the instantaneous measurement of muscle metabolism. Proc R Soc Lond [Biol]. 1937:218–41.

[52] Wittenberg B, Wittenberg J, Caldwell P (1975). Role of myoglobin in the oxygen supply to red skeletal muscle. J Biol Chem.

[53] Reynafarje B (1962). Myoglobin content and enzymatic activity of muscle and altitude adaptation. J Appl Physiol.

[54] Helbo S, Fago A (2012). Functional properties of myoglobins from five whale species with different diving capacities. J Exp Biol.

[55] Driedzic WR, Stewart JM, Scott DL (1982). The protective effect of myoglobin during hypoxic perfusion of isolated fish hearts. J Mol Cell Cardiol.

[56] Acierno R, Agnisola C, Tota B, Sidell B (1997). Myoglobin enhances cardiac performance in antarctic icefish species that express the protein. Am J Physiol Regul Integr Comp Physiol.

[57] Brunori M (2001). Nitric oxide moves myoglobin Centre stage. Trends Biochem Sci.

[58] Flögel U, Gödecke A, Klotz L-O, Schrader J (2004). Role of myoglobin in the antioxidant defense of the heart. FASEB J.

[59] Li C, Jackson RM (2002). Reactive species mechanisms of cellular hypoxia-reoxygenation injury. Am J Physiol Cell Physiol.

[60] Bickler PE, Buck LT (2007). Hypoxia tolerance in reptiles, amphibians, and fishes: life with variable oxygen availability. Annu Rev Physiol.

[61] Helbo S, Dewilde S, Williams DR, Berghmans H, Berenbrink M, Cossins AR, Fago A (2012). Functional differentiation of myoglobin isoforms in hypoxia-tolerant carp indicates tissue-specific protective roles. Am J Physiol Regul Integr Comp Physiol..

[62] Bargmann CI (1997). Olfactory receptors, vomeronasal receptors, and the organization of olfactory information. Cell.

[63] Mombaerts P (2004). Genes and ligands for odorant, vomeronasal and taste receptors. Nat Rev Neurosci.

[64] Yoshihara Y, Meyerhof W, Korsching SI (2008). Molecular genetic dissection of the zebrafish olfactory system. Chemosensory Systems in Mammals, fishes, and insects. Springer.

[65] Cao Y, Oh BC, Stryer L (1998). Cloning and localization of two multigene receptor families in goldfish olfactory epithelium. Proc Natl Acad Sci U S A.

[66] Pfister P, Rodriguez I (2005). Olfactory expression of a single and highly variable V1r pheromone receptor-like gene in fish species. Proc Natl Acad Sci U S A.

[67] Young JM, Massa HF, Hsu L, Trask BJ (2010). Extreme variability among mammalian V1R gene families. Genome Res.

[68] Kurzweil VC, Getman M, Green ED, Lane RP (2009). Dynamic evolution of V1R putative pheromone receptors between *Mus musculus* and *Mus spretus*. BMC Genomics.

[69] Syed AS, Korsching SI (2014). Positive Darwinian selection in the singularly large taste receptor gene family of an ‘ancient’fish, *Latimeria chalumnae*. BMC Genomics.

[70] Nikaido M, Noguchi H, Nishihara H, Toyoda A, Suzuki Y, Kajitani R, Suzuki H, Okuno M, Aibara M, Ngatunga BP (2013). Coelacanth genomes reveal signatures for evolutionary transition from water to land. Genome Res.

[71] Van der Oost R, Beyer J, Vermeulen NP (2003). Fish bioaccumulation and biomarkers in environmental risk assessment: a review. Environ Toxicol Pharmacol.

[72] Mulder GJ (2003). Conjugation reactions in drug metabolism: an integrated approach.

[73] Farah MA, Ateeq B, Ali MN, Sabir R, Ahmad W (2004). Studies on lethal concentrations and toxicity stress of some xenobiotics on aquatic organisms. Chemosphere.

[74] Mos L, Cooper GA, Serben K, Cameron M, Koop BF (2008). Effects of diesel on survival, growth, and gene expression in rainbow trout (*Oncorhynchus mykiss*) fry. Environ Sci Technol.

[75] Zhu L, Qu K, Xia B, Sun X, Chen B (2016). Transcriptomic response to water accommodated fraction of crude oil exposure in the gill of Japanese flounder, *Paralichthys olivaceus*. Marine Poll Bull.

[76] Martin K (2014). Theme and variations: amphibious air-breathing intertidal fishes. J Fish Biol.

[77] Janis CM, Farmer C (1999). Proposed habitats of early tetrapods: gills, kidneys, and the water-land transition. Zool J Linnean Soc.

[78] Randall DJ (1981). The evolution of air breathing in vertebrates.

[79] Romer AS (1967). Major steps in vertebrate evolution. Science.

[80] Inger RF (1957). Ecological aspects of the origins of the tetrapods. Evolution.

[81] Henry RP, Heming TA (1998). Carbonic anhydrase and respiratory gas exchange. Fish Physiol.

[82] Randall D, Val A, Heisler N (1995). The role of carbonic anhydrase in aquatic gas exchange. Mechanisms of Systemic Regulation Springer.

[83] Gilmour K, Perry S (2009). Carbonic anhydrase and acid–base regulation in fish. J Exp Biol.

[84] Ahmed A, Mohamed K, Ahmed S-A, Masoud F (2008). Anatomical, light and scanning electron microscopic studies on the air breathing dendretic organ of the sharp tooth catfish (*Clarias gariepinus*). J Vet Anat.

[85] Ikpegbu E, Nlebedum U, Nnadozie O, Agbakwuru O (2013). Histological observations on the dendretic organ of the farmed adult African catfish (*Clarias gariepinus*) from eastern Nigeria. J Agric Sci.

[86] Luo W, Cao X, Xu X, Huang S, Liu C, Tomljanovic T (2016). Developmental transcriptome analysis and identification of genes involved in formation of intestinal air-breathing function of dojo loach, *Misgurnus anguillicaudatus*. Sci Rep.

[87] Jiang Y, Feng S, Xu J, Zhang S, Li S, Sun X, Xu P (2016). Comparative transcriptome analysis between aquatic and aerial breathing organs of *Channa argus* to reveal the genetic basis underlying bimodal respiration. Mar Genomics.

[88] Chandra S, Banerjee TK (2003). Histopathological analysis of the respiratory organs of the air-breathing catfish *Clarias batrachus* (Linn.) exposed to the air. Acta Zool Taiwanica.

[89] Andrews S. FastQC: A quality control tool for high throughput sequence data*.* 2010. (Available online at: http://www.bioinformatics.babraham.ac.uk/projects/fastqc).

[90] Martin M (2011). Cutadapt removes adapter sequences from high-throughput sequencing reads. EMBnet J.

[91] Simpson JT, Wong K, Jackman SD, Schein JE, Jones SJ, Birol I (2009). ABySS: a parallel assembler for short read sequence data. Genome Res.

[92] Gnerre S, MacCallum I, Przybylski D, Ribeiro FJ, Burton JN, Walker BJ, Sharpe T, Hall G, Shea TP, Sykes S (2011). High-quality draft assemblies of mammalian genomes from massively parallel sequence data. Proc Natl Acad Sci U S A.

[93] Boetzer M, Henkel CV, Jansen HJ, Butler D, Pirovano W (2011). Scaffolding pre-assembled contigs using SSPACE. Bioinformatics.

[94] Boetzer M, Pirovano W (2012). Toward almost closed genomes with GapFiller. Genome Biol.

[95] Delcher AL, Salzberg SL, Phillippy AM. Using MUMmer to identify similar regions in large sequence sets. Curr Protoc Bioinformatics. 2003; chapter 10, unit 10.3. 10.1002/0471250953.bi1003s00 18428693

[96] Bao Z, Eddy SR (2002). Automated *de novo* identification of repeat sequence families in sequenced genomes. Genome Res.

[97] Price AL, Jones NC, Pevzner PA (2005). *De novo* identification of repeat families in large genomes. Bioinformatics.

[98] Jukes TH, Cantor CR (1969). Evolution of protein molecules. Mammalian Protein Metabolism.

[99] Stanke M, Steinkamp R, Waack S, Morgenstern B (2004). AUGUSTUS: a web server for gene finding in eukaryotes. Nucleic Acids Res.

[100] Conesa A, Götz S, García-Gómez JM, Terol J, Talón M, Robles M (2005). Blast2GO: a universal tool for annotation, visualization and analysis in functional genomics research. Bioinformatics.

[101] Moriya Y, Itoh M, Okuda S, Yoshizawa AC, Kanehisa M (2007). KAAS: an automatic genome annotation and pathway reconstruction server. Nucleic Acids Res.

[102] Emms D, Kelly S (2015). OrthoFinder: solving fundamental biases in whole genome comparisons dramatically improves orthogroup inference accuracy. Genome Biol.

[103] Mi H, Huang X, Muruganujan A, Tang H, Mills C, Kang D, Thomas PD (2016). PANTHER version 11: expanded annotation data from gene ontology and Reactome pathways, and data analysis tool enhancements. Nucleic Acids Res.

[104] Edgar RC (2004). MUSCLE: multiple sequence alignment with high accuracy and high throughput. Nucleic Acids Res.

[105] Suyama M, Torrents D, Bork P (2006). PAL2NAL: robust conversion of protein sequence alignments into the corresponding codon alignments. Nucleic Acids Res.

[106] Talavera G, Castresana J (2007). Improvement of phylogenies after removing divergent and ambiguously aligned blocks from protein sequence alignments. Syst Biol.

[107] Borowiec ML (2016). AMAS: a fast tool for alignment manipulation and computing of summary statistics. PeerJ.

[108] Lanfear R, Frandsen PB, Wright AM, Senfeld T, Calcott B (2016). PartitionFinder 2: new methods for selecting partitioned models of evolution for molecular and morphological phylogenetic analyses. Mol Biol Evol.

[109] Stamatakis A (2014). RAxML version 8: a tool for phylogenetic analysis and post-analysis of large phylogenies. Bioinformatics.

[110] Yang Z (2007). PAML 4: phylogenetic analysis by maximum likelihood. Mol Biol Evol.

[111] Gojobori T (1983). Codon substitution in evolution and the “saturation” of synonymous changes. Genetics.

[112] Zhang J, Nielsen R, Yang Z (2005). Evaluation of an improved branch-site likelihood method for detecting positive selection at the molecular level. Mol Biol Evol.

[113] Yang Z, Wong WS, Nielsen R (2005). Bayes empirical Bayes inference of amino acid sites under positive selection. Mol Biol Evol.

[114] Kinsella RJ, Kähäri A, Haider S, Zamora J, Proctor G, Spudich G, Almeida-King J, Staines D, Derwent P, Kerhornou A (2011). Ensembl BioMarts: a hub for data retrieval across taxonomic space. Database.

[115] Fabregat A, Sidiropoulos K, Garapati P, Gillespie M, Hausmann K, Haw R, Jassal B, Jupe S, Korninger F, McKay S (2016). The reactome pathway knowledgebase. Nucleic Acids Res.

[116] Han MV, Thomas GW, Lugo-Martinez J, Hahn MW (2013). Estimating gene gain and loss rates in the presence of error in genome assembly and annotation using CAFE 3. Mol Biol Evol.

[117] Thompson JD, Gibson T, Higgins DG. Multiple sequence alignment using ClustalW and ClustalX. Curr Protoc Bioinformatics. 2003;(1):2–3. 10.1002/0471250953.bi0203s00 18792934

[118] Tamura K, Stecher G, Peterson D, Filipski A, Kumar S (2013). MEGA6: molecular evolutionary genetics analysis version 6.0. Mol Biol Evol.

[119] Darriba D, Taboada GL, Doallo R, Posada D (2011). ProtTest 3: fast selection of best-fit models of protein evolution. Bioinformatics.

[120] Trapnell C, Williams BA, Pertea G, Mortazavi A, Kwan G, Van Baren MJ, Salzberg SL, Wold BJ, Pachter L (2010). Transcript assembly and quantification by RNA-Seq reveals unannotated transcripts and isoform switching during cell differentiation. Nat Biotechnol.

[121] Trapnell C, Pachter L, Salzberg SL (2009). TopHat: discovering splice junctions with RNA-Seq. Bioinformatics.

[122] Trapnell C, Roberts A, Goff L, Pertea G, Kim D, Kelley DR, Pimentel H, Salzberg SL, Rinn JL, Pachter L (2012). Differential gene and transcript expression analysis of RNA-seq experiments with TopHat and cufflinks. Nat Protoc.

[123] Bolger AM, Lohse M, Usadel B (2014). Trimmomatic: a flexible trimmer for Illumina sequence data. Bioinformatics.

[124] CLC Genomics Workbench (https://www.qiagenbioinformatics.com/).

[125] Kal AJ, van Zonneveld AJ, Benes V, van den Berg M, Koerkamp MG, Albermann K, Strack N, Ruijter JM, Richter A, Dujon B (1999). Dynamics of gene expression revealed by comparison of serial analysis of gene expression transcript profiles from yeast grown on two different carbon sources. Mol Biol Cell.

[126] Mohindra V, Tripathi RK, Singh A, Singh RK, Lal KK (2014). Identification of candidate reference genes for quantitative expression analysis by real-time PCR for hypoxic stress in Indian catfish, *Clarias batrachus* (Linnaeus, 1758). Int Aquat Res.

[127] Pfaffl MW, Horgan GW, Dempfle L (2002). Relative expression software tool (REST) for group-wise comparison and statistical analysis of relative expression results in real-time PCR. Nucleic Acids Res.

