# Supplementary material for: Genome sequence of walking catfish (Clarias batrachus) provides insights into terrestrial adaptation
Source: BMC Genomics. 2018 Dec 20;19:952. doi: 10.1186/s12864-018-5355-9 (PMC6302426; doi:10.1186/s12864-018-5355-9)
Supplement: Supplementary file 1 — Figure S1. Multiple sequence alignment of myoglobin genes in the genomes of Clarias batrachus, Danio rerio, Ictalurus punctatus and Astyanax mexicanus. Figure S2. Detailed phylogenetic tree of olfactory receptor related to class A G protein-coupled receptor (ora) with species names and sequence names for Figure 3b. Figure S3. Multiple sequence alignment of olfactory receptor related to class A G protein-coupled receptor 1 (ora1) in the genomes of Clarias batrachus, Danio rerio, Ictalurus punctatus and Astyanax mexicanus. Figure S4. Syntenic analysis of myoglobin gene (mb) using genomic information of Danio rerio, Ictalurus punctatus and Clarias batrachus. Figure S5. Comparison of relative fold changes between air-breathing organ and gill in Clarias batrachus after normalization to 28S rRNA using RNA-Seq datasets and qRT-PCR. Table S1. Completeness of genome assembly assessed by CEGMA and BUSCO. Table S2. Mapping of five longest ABySS scaffolds to genome assembly. Table S3. Repetitive elements in the Clarias batrachus genome. Table S4. Orthogroups shared between species by OrthoFinder. Table S5. Specific genes in the Clarias batrachus genome compared with that of channel catfish (Ictalurus punctatus). Table S6. GO terms significantly enriched in the specific genes in the Clarias batrachus genome compared with that of channel catfish (Ictalurus punctatus). Table S7. Positively selected genes in the Clarias batrachus genome compared with those of 11 non-air-breathing teleost fish. Table S8. GO terms significantly enriched in the positively selected genes in the Clarias batrachus genome. Table S9. Expanded genes in the Clarias batrachus genome. Table S10. Summary of transcriptome sequencing data. Table S11. Expression values (RPKM) of all the genes in the transcriptomes of the gill and the air-breathing organ. Table S12. Differentially expressed genes in the transcriptome of the air-breathing organ compared with that of the gill. Table S13. GO terms significantly enrich [file 12864_2018_5355_MOESM1_ESM.zip › supplementary files.docx]

**Supplementary Files**

**
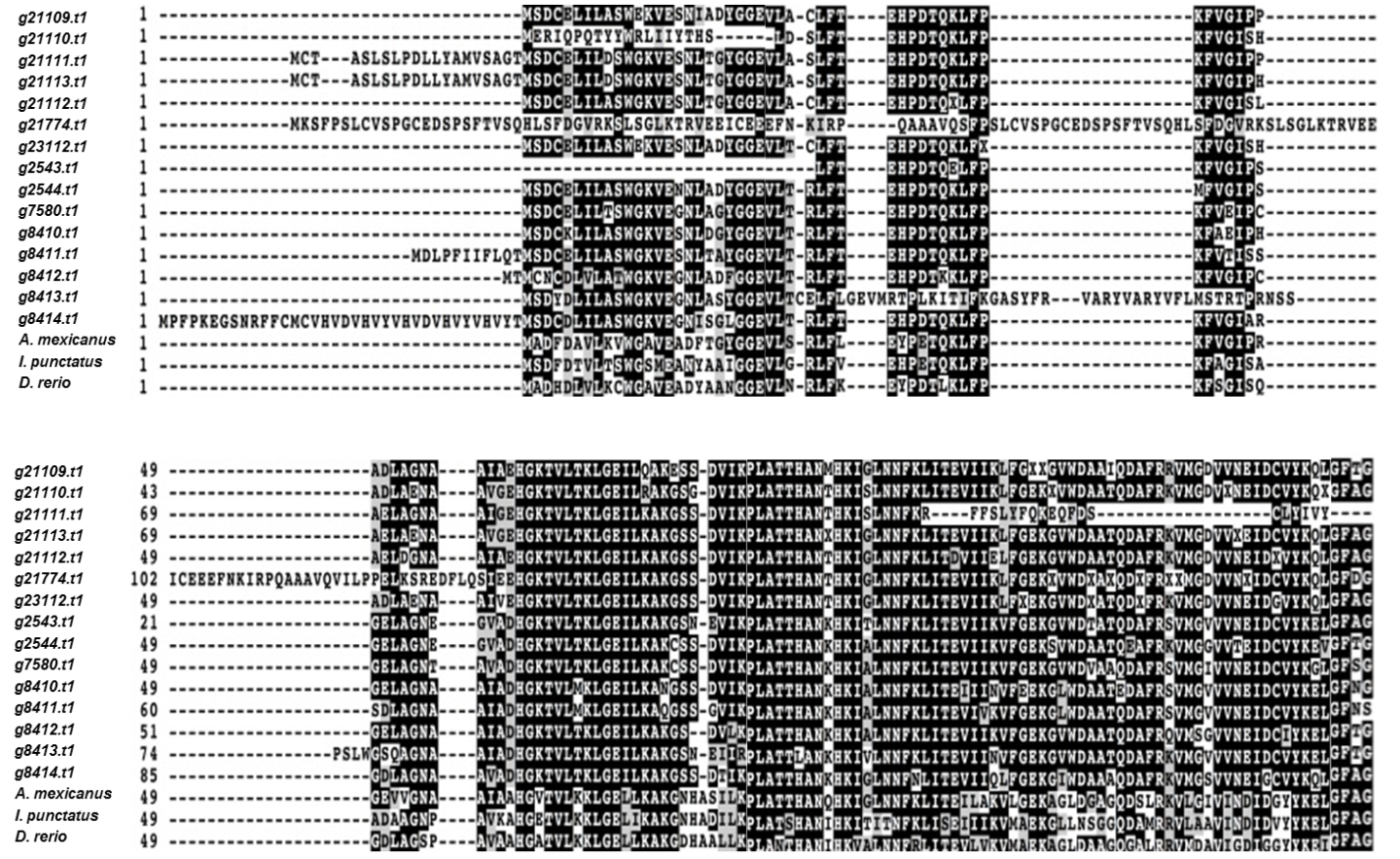
**

**Supplementary Figure 1.** **Multiple sequence alignment of myoglobin genes in the genomes of *Clarias batrachus*, *Danio rerio*, *Ictalurus punctatus* and *Astyanax mexicanus*.**

**
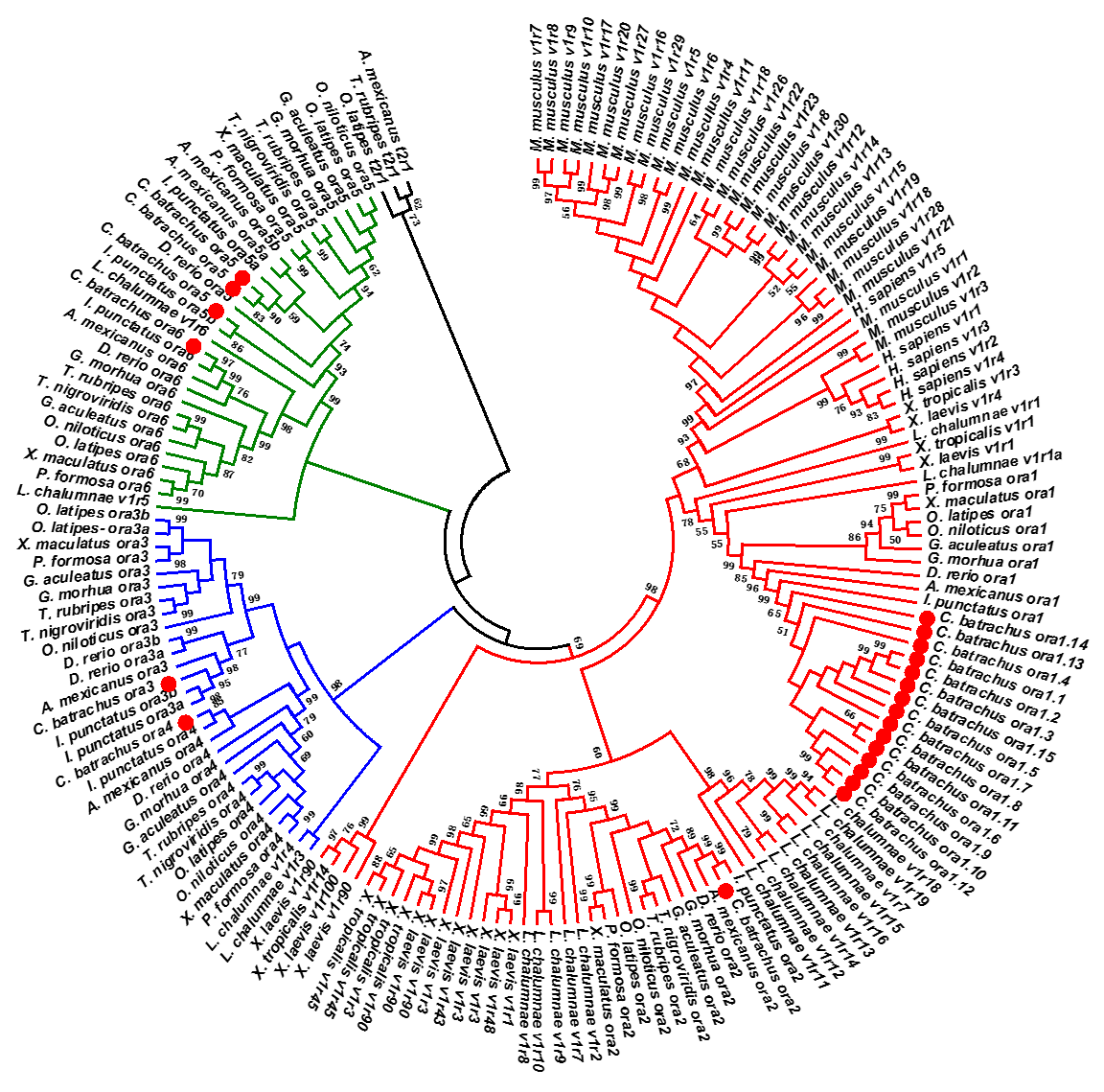
**

**Supplementary Figure 2. Detailed phylogenetic tree of olfactory receptor related to class A G protein-coupled receptor (*ora*) with species names and sequence names for Figure 3b.**

Three clades are indicated with red (*ora1*-*ora2* clade), blue (*ora3*-*ora4* clade) and green (*ora5*-*ora6* clade). The red solid circles represent the *ora* genes in the *Clarias batrachus* genome. Bootstrap support (1,000 replications) is indicated on the nodes. The accession numbers of protein sequences and models used in the construction of phylogenetic tree are provided in Supplementary Table 15.


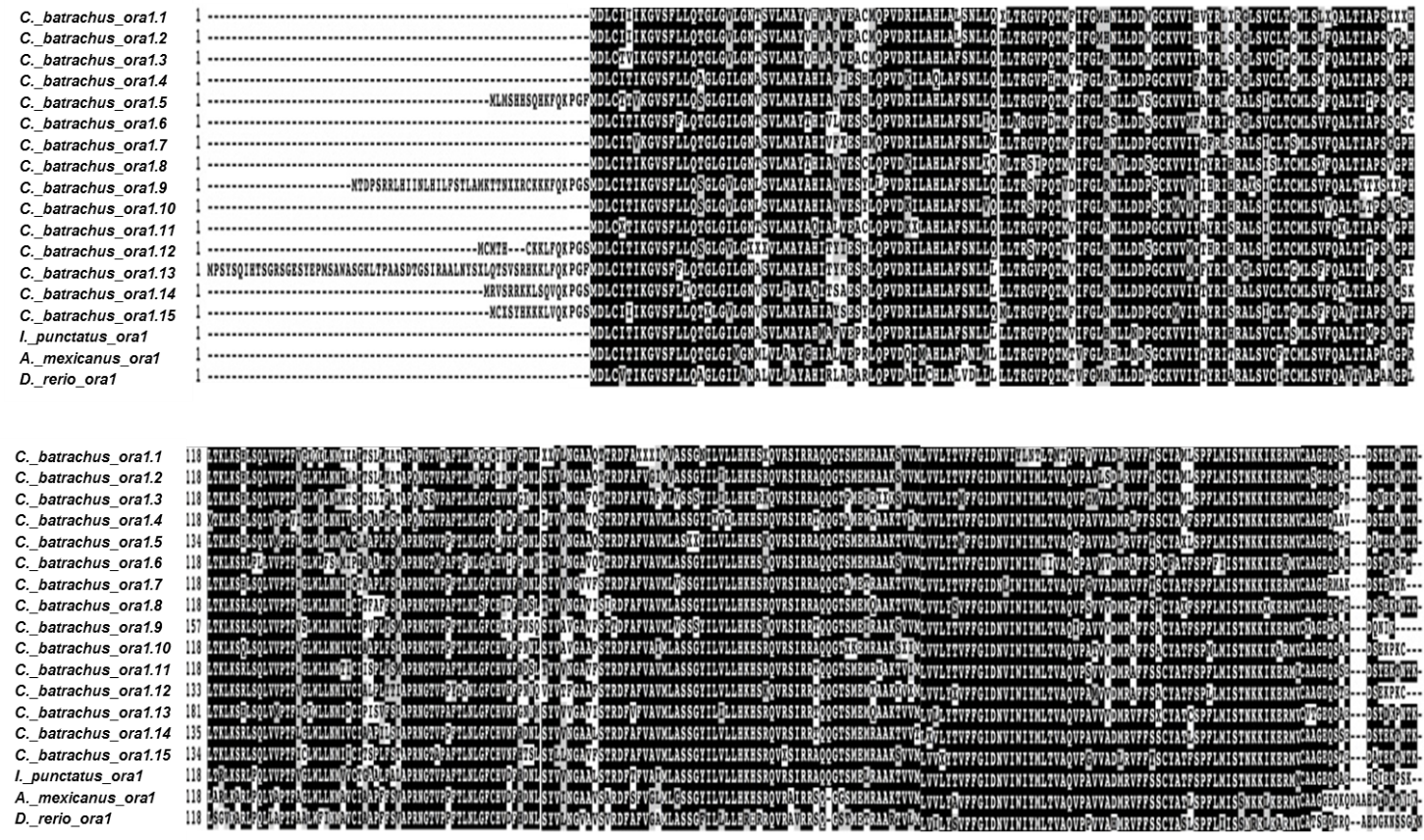


**Supplementary Figure 3. Multiple sequence alignment of olfactory receptor related to class A G protein-coupled receptor 1 (*ora1*) in the genomes of *Clarias batrachus*, *Danio rerio*, *Ictalurus punctatus* and *Astyanax mexicanus*.**


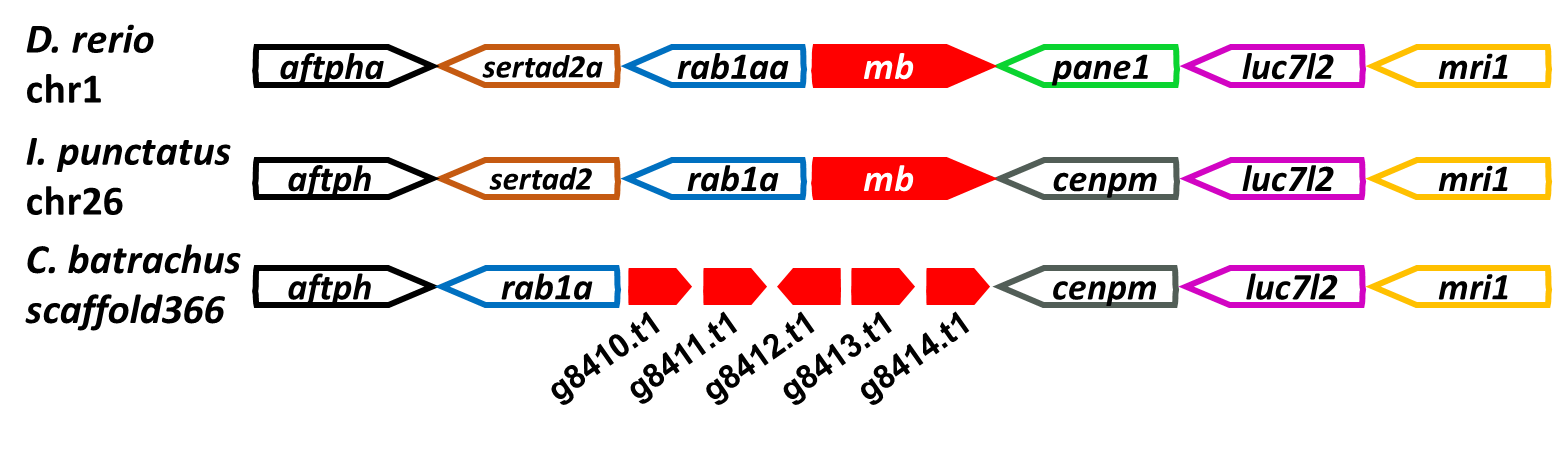


**Supplementary Figure 4.** **Syntenic analysis of myoglobin gene (*mb*) using genomic information of *Danio rerio*, *Ictalurus punctatus* and *Clarias batrachus*.**

The g8410.t1, g8411.t1, g8412.t1, g8413.t1 and g8414.t1 represent the myoglobin genes on the scaffold366 in the *C. batrachus* genome. *aftph*, aftiphilin; *aftpha*, aftiphilin a; *sertad2*, SERTA domain containing 2; *sertad2a*, SERTA domain containing 2a; *rab1a*, RAB1A, member RAS oncogene family; *rab1aa*, RAB1A, member RAS oncogene family a; *pane1*, proliferation associated nuclear element; *cenpm*, centromere protein M; *luc7l2*, putative RNA-binding protein Luc7-like 2; *mri1*, methylthioribose-1-phosphate isomerase 1.


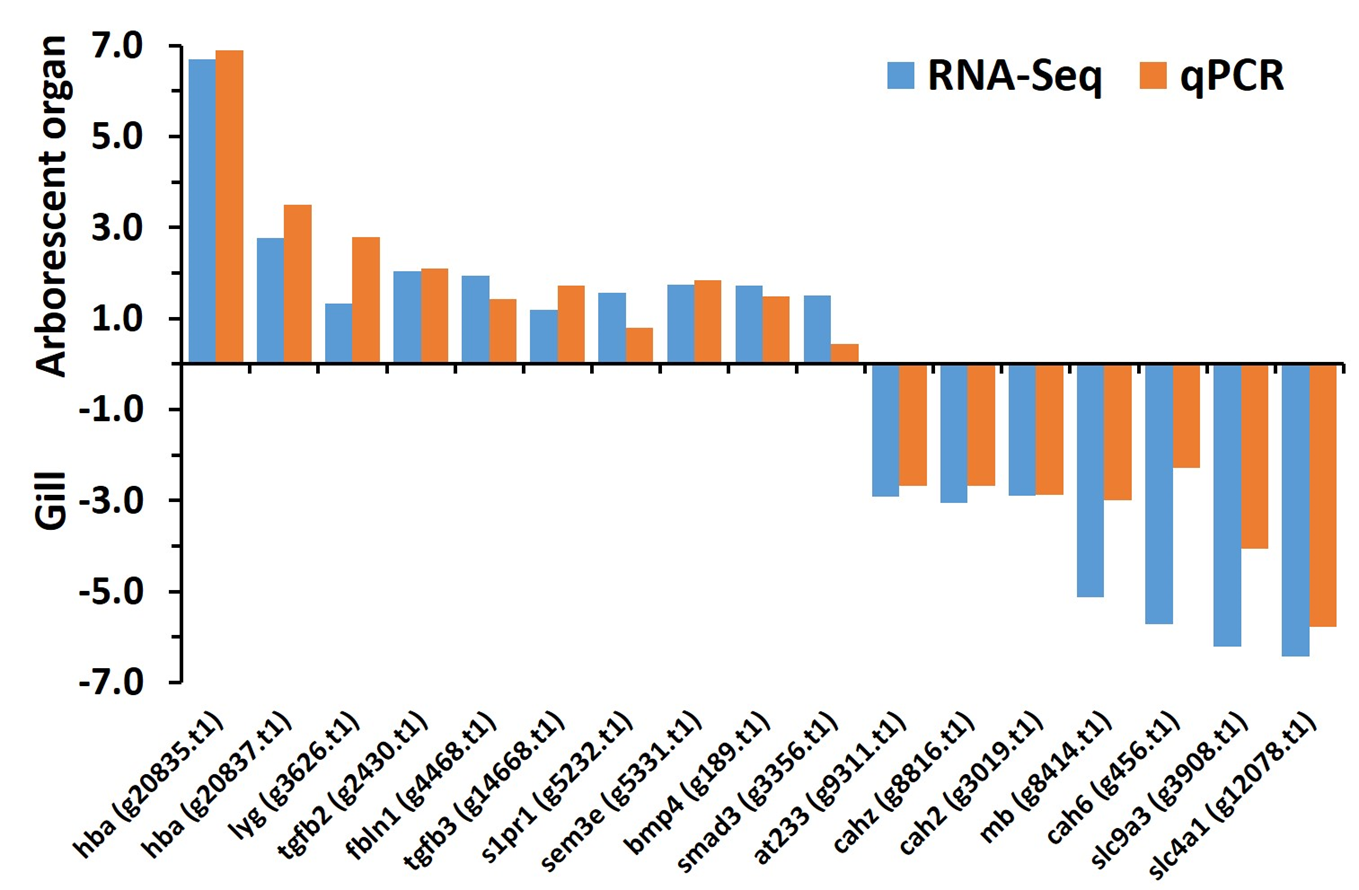


**Supplementary Figure 5. Comparison of relative fold changes between air-breathing organ and gill in *Clarias batrachus* after normalization to 28S rRNA using RNA-Seq datasets and qRT-PCR.**

The absolute values of relative fold changes were transformed by log2(Ratio). The Pearson correlation coefficient between the two results is 0.8944 before transformation.

**Supplementary Table 1. Completeness of genome assembly assessed by CEGMA and BUSCO.**

|  | **Complete orthologues (%)** | **Partial orthologues (%)** | **# Total orthologues analyzed** |
| --- | --- | --- | --- |
| **CEGMA** | 73.4 | 21.8 | 248 |
| **BUSCO** | 72.8 | 11.1 | 3,023 |

**Supplementary** **Table 2. Mapping of five longest ABySS scaffolds to genome assembly.**

| **ABySS**  **scaffold ID** | **ABySS**  **aligned length (bp)** | **scaffold ID** | **Aligned length (bp)** | **Aligned identity (%)** |
| --- | --- | --- | --- | --- |
| 3829531 | 2,275,460 | scaffold7;  scaffold192 | 2,273,636 | 99.77 |
| 3833592 | 1,609,354 | scaffold107;  scaffold129 | 1,607,455 | 99.78 |
| 3829461 | 1,444,754 | scaffold2 | 1,442,715 | 99.54 |
| 3821019 | 1,565,771 | scaffold8 | 1,564,193 | 99.75 |
| 3829340 | 1,371,683 | scaffold17 | 1,370,520 | 99.43 |

**Supplementary Table 3. Repetitive elements in the *Clarias batrachus* genome.**

| **Repetitive elements** | **Number of elements** | **Length occupied (bp)** | **Percentage of genome (%)** |
| --- | --- | --- | --- |
| **Interspersed repeats** |  |  |  |
| SINEs | 61,630 | 9,458,516 | 1.15 |
| LINEs | 110,499 | 27,867,994 | 3.39 |
| LTR elements | 113,379 | 30,149,843 | 3.67 |
| DNA elements | 577,729 | 126,278,979 | 15.37 |
| Unclassified | 292,027 | 54,288,506 | 6.61 |
| *Subtotal* | *1,155,264* | *248,043,838* | *30.18* |
| **Tandem repeats** |  |  |  |
| Satellites | 2,097 | 624,424 | 0.08 |
| Simple repeats | 1,516 | 135,706 | 0.02 |
| *Subtotal* | *3,613* | *760,130* | *0.1* |
| **Total** | **1,158,877** | **248,803,960** | **30.28** |

**Supplementary Table 4. Orthogroups shared between species by OrthoFinder.**

|  | ***Poecilia formosa*** | ***Astyanax mexicanus*** | ***Ictalurus punctatus*** | ***Clarias batrachus*** | ***Gadus morhua*** | ***Takifugu rubripes*** | ***Oryzias latipes*** | ***Xiphophorus maculatus*** | ***Lepisosteus oculatus*** | ***Gasterosteus aculeatus*** | ***Tetraodon nigroviridis*** | ***Oreochromis niloticus*** |
| --- | --- | --- | --- | --- | --- | --- | --- | --- | --- | --- | --- | --- |
| ***Astyanax mexicanus*** | 11,157 |  |  |  |  |  |  |  |  |  |  |  |
| ***Ictalurus punctatus*** | 11,578 | 12,986 |  |  |  |  |  |  |  |  |  |  |
| ***Clarias batrachus*** | 10,613 | 11,941 | 12,806 |  |  |  |  |  |  |  |  |  |
| ***Gadus morhua*** | 10,969 | 11,684 | 12,184 | 11,205 |  |  |  |  |  |  |  |  |
| ***Takifugu rubripes*** | 10,751 | 11,471 | 11,934 | 10,973 | 11,509 |  |  |  |  |  |  |  |
| ***Oryzias latipes*** | 10,887 | 11,466 | 11,920 | 10,976 | 11,429 | 11,323 |  |  |  |  |  |  |
| ***Xiphophorus maculatus*** | 11,742 | 12,016 | 12,448 | 11,380 | 11,863 | 11,660 | 11,675 |  |  |  |  |  |
| ***Lepisosteus oculatus*** | 10,821 | 11,850 | 12,403 | 11,285 | 11,332 | 11,118 | 11,084 | 11,586 |  |  |  |  |
| ***Gasterosteus aculeatus*** | 11,328 | 11,958 | 12,454 | 11,415 | 12,556 | 11,793 | 11,774 | 12,256 | 11,557 |  |  |  |
| ***Tetraodon nigroviridis*** | 10,749 | 11,494 | 11,935 | 11,010 | 11,448 | 11,652 | 11,356 | 11,648 | 11,087 | 11,730 |  |  |
| ***Oreochromis niloticus*** | 11,151 | 11,794 | 12,210 | 11,191 | 11,587 | 11,582 | 11,472 | 12,056 | 11,410 | 11,933 | 11,478 |  |
| ***Danio rerio*** | 11,020 | 12,216 | 12,822 | 11,642 | 11,543 | 11,266 | 11,298 | 11,799 | 11,694 | 11,792 | 11,278 | 11,594 |

**Supplementary Table 5. Specific genes in the *Clarias batrachus* genome compared with that of channel catfish (*Ictalurus* *punctatus*).**

(Included in a separated excel file)

**Supplementary Table 6. GO terms significantly enriched in the specific genes in the *Clarias batrachus* genome compared with that of channel catfish (*Ictalurus punctatus*).**

| **GO ID** | **GO Term** | **Category** | **Bonferroni-corrected *P*-value** |
| --- | --- | --- | --- |
| GO:0006281 | DNA repair | BP | 1.66E-04 |
| GO:0006259 | DNA metabolic process | BP | 1.76E-03 |
| GO:0009987 | cellular process | BP | 1.52E-04 |
| GO:0008047 | enzyme activator activity | MF | 1.19E-02 |
| GO:0030234 | enzyme regulator activity | MF | 2.63E-05 |
| GO:0005085 | guanyl-nucleotide exchange factor activity | MF | 6.52E-03 |
| GO:0005083 | small GTPase regulator activity | MF | 5.65E-06 |
| GO:0030054 | cell junction | CC | 3.85E-02 |

**Supplementary Table 7. Positively selected genes in the *Clarias batrachus* genome compared with those of 11 non-air-breathing teleost fish.**

(Included in a separated excel file)

**Supplementary Table 8. GO terms significantly enriched in the positively selected genes in the *Clarias batrachus* genome.**

| **GO ID** | **GO Term** | **Category** | **FDR** |
| --- | --- | --- | --- |
| GO:0070013 | intracellular organelle lumen | CC | 5.30E-08 |
| GO:0031974 | membrane-enclosed lumen | CC | 5.30E-08 |
| GO:0043233 | organelle lumen | CC | 5.30E-08 |
| GO:0044446 | intracellular organelle part | CC | 5.30E-08 |
| GO:0044422 | organelle part | CC | 1.16E-07 |
| GO:0044428 | nuclear part | CC | 5.82E-06 |
| GO:0043231 | intracellular membrane-bounded organelle | CC | 3.44E-05 |
| GO:0043227 | membrane-bounded organelle | CC | 1.23E-04 |
| GO:0032991 | macromolecular complex | CC | 1.74E-04 |
| GO:0031981 | nuclear lumen | CC | 1.01E-03 |
| GO:0043229 | intracellular organelle | CC | 1.80E-03 |
| GO:0044424 | intracellular part | CC | 2.03E-03 |
| GO:0005739 | mitochondrion | CC | 2.71E-03 |
| GO:0043226 | organelle | CC | 3.93E-03 |
| GO:0044429 | mitochondrial part | CC | 3.93E-03 |
| GO:0005758 | mitochondrial intermembrane space | CC | 6.08E-03 |
| GO:0044451 | nucleoplasm part | CC | 6.42E-03 |
| GO:0031970 | organelle envelope lumen | CC | 6.65E-03 |
| GO:0090575 | RNA polymerase II transcription factor complex | CC | 6.99E-03 |
| GO:0043234 | protein complex | CC | 8.40E-03 |
| GO:0044798 | nuclear transcription factor complex | CC | 1.18E-02 |
| GO:0005634 | nucleus | CC | 1.63E-02 |
| GO:0005654 | nucleoplasm | CC | 1.79E-02 |
| GO:0005622 | intracellular | CC | 3.13E-02 |
| GO:0044444 | cytoplasmic part | CC | 3.47E-02 |
| GO:0034641 | cellular nitrogen compound metabolic process | BP | 4.46E-02 |
| GO:0055029 | nuclear DNA-directed RNA polymerase complex | CC | 4.58E-02 |
| GO:0030880 | RNA polymerase complex | CC | 4.58E-02 |
| GO:0000428 | DNA-directed RNA polymerase complex | CC | 4.58E-02 |

**Supplementary Table 9. Expanded genes in the *Clarias batrachus* genome.**

| **Gene name** | **Gene list** |
| --- | --- |
| *mb* | g21109.t1, g21110.t1, g21111.t1, g21112.t1, g21113.t1, g21774.t1, g23112.t1, g2543.t1, g2544.t1, g7580.t1, g8410.t1, g8411.t1, g8412.t1, g8413.t1, g8414.t1 |
| *ora1* | g18228.t1, g18229.t1, g18230.t1, g18231.t1, g18232.t1, g18233.t1, g18234.t1, g18235.t1, g18236.t1, g18237.t1, g18238.t1, g18239.t1, g18240.t1, g18241.t1 |
| *sult6b1* | g16408.t1, g20979.t1, g20980.t1, g20981.t1, g20982.t1, g20983.t1, g20984.t1, g20985.t1, g20986.t1, g24152.t1, g24341.t1, g24380.t1 |

**Supplementary** **Table 10.** **Summary of transcriptome sequencing data.**

| **Tissue** | **Read length (bp)** | **# of raw reads** | **Raw data (Gb)** | **# of trimmed reads** |
| --- | --- | --- | --- | --- |
| **Gill** | 125 | 234,620,438 | 29.3 | 225,509,720 |
| **Air-breathing organ** | 125 | 231,374,100 | 28.9 | 221,641,474 |

**Supplementary Table 11. Expression values (RPKM) of all the genes in the transcriptomes of the gill and the air-breathing organ.**

(Included in a separated excel file)

**Supplementary Table 12. Differentially expressed genes in the transcriptome of the air-breathing organ compared with that of the gill.**

(Included in a separated excel file)

**Supplementary Table 13. GO terms significantly enriched in the differentially expressed genes comparing the transcriptome of the air-breathing organ with that of the gill.**

(Included in a separated excel file)

**Supplementary Table 14. Pathways significantly enriched in the differentially expressed genes comparing the transcriptome of the air-breathing organs with that of the gill.**

|  | **Pathway name** | ***P*-value** |
| --- | --- | --- |
| **Up-regulated** | Formation of the cornified envelope | 6.73E-07 |
|  | Molecules associated with elastic fibres | 2.30E-04 |
|  | Keratinization | 4.94E-04 |
|  | Elastic fibre formation | 6.25E-04 |
|  | The activation of arylsulfatases | 8.20E-04 |
|  | Synthesis of 15-eicosatetraenoic acid derivatives | 9.56E-04 |
|  | Synthesis of 12-eicosatetraenoic acid derivatives | 9.56E-04 |
|  | Syndecan interactions | 1.77E-03 |
|  | Signaling by FGFR1 amplification mutants | 1.88E-03 |
|  | Hydrolysis of LPC | 2.16E-03 |
|  | Erythrocytes take up oxygen and release carbon dioxide | 2.16E-03 |
|  | Arachidonic acid metabolism | 2.62E-03 |
|  | Extracellular matrix organization | 2.81E-03 |
|  | Interleukin-4 and 13 signaling | 3.00E-03 |
|  | Phospholipase C-mediated cascade; FGFR2 | 4.59E-03 |
|  | Acyl chain remodelling of PS | 5.40E-03 |
|  | FGFR2 ligand binding and activation | 6.30E-03 |
|  | Signaling by FGFR2 IIIa TM | 6.30E-03 |
|  | O2/CO2 exchange in erythrocytes | 6.67E-03 |
|  | Erythrocytes take up carbon dioxide and release oxygen | 6.67E-03 |
|  | TP53 Regulates Transcription of Death Receptors and Ligands | 6.67E-03 |
|  | Constitutive Signaling by Aberrant PI3K in Cancer | 9.51E-03 |
|  | PI-3K cascade:FGFR2 | 9.56E-03 |
| **Down-regulated** | Keratinization | 1.11E-16 |
|  | Formation of the cornified envelope | 1.11E-16 |
|  | Striated Muscle Contraction | 9.81E-13 |
|  | Muscle contraction | 1.45E-06 |
|  | Reversible hydration of carbon dioxide | 1.72E-05 |
|  | Apoptotic cleavage of cell adhesion proteins | 1.36E-04 |
|  | Apoptotic cleavage of cellular proteins | 3.04E-04 |
|  | Erythrocytes take up oxygen and release carbon dioxide | 4.53E-04 |
|  | Apoptotic execution phase | 5.35E-04 |
|  | Glycolysis | 8.16E-04 |
|  | O2/CO2 exchange in erythrocytes | 2.00E-03 |
|  | Erythrocytes take up carbon dioxide and release oxygen | 2.00E-03 |
|  | Branched-chain amino acid catabolism | 3.57E-03 |
|  | Pyruvate metabolism and Citric Acid (TCA) cycle | 5.47E-03 |
|  | Rhesus glycoproteins mediate ammonium transport | 7.75E-03 |
|  | Ion homeostasis | 1.00E-02 |

**Supplementary Table 15. Accession numbers of protein sequences and models used in the construction of phylogenetic trees of expanded genes.**

(Included in a separated excel file)

**Supplementary Table 16. Primer sequences used for qRT-PCR.**

| **Gene name** | **Gene ID** | **Forward primer** | **Reverse primer** |
| --- | --- | --- | --- |
| *28S rRNA* | JK488212 | 5' TTTCAGGGCTAGTTGATTCGGCAG | 5' GGTTGATATAGACAGCAGGACGGT |
| *hba* | g20835.t1 | 5' ACAAGGCCGTTGTGAAAGAC | 5' GCCAGTGAGCGAAGTAGGTC |
| *hba* | g20837.t1 | 5' ACAAGGCCGTTGTGAAGGAG | 5' CCCAGTGAGCGAAGTAGGTC |
| *lyg* | g3626.t1 | 5' GTCACCCAAGGCACTGAAAT | 5' TAGGCTGAGATTCCCCCTTT |
| *tgfb2* | g2430.t1 | 5' GCTTTGCAGGGATTGATGAT | 5' TCTGTCTGTGGCTCTTGTGC |
| *fbln1* | g4468.t1 | 5' ACAGGCGAGCAAGTGAAACT | 5' ACAGGGATGTTGATGGGAAA |
| *tgfb3* | g14668.t1 | 5' CATCGATTTCCGACAGGACT | 5' AGCGAACTGTGTGTGGTGTC |
| *s1pr1* | g5232.t1 | 5' GAAATTCCGCAAAGTGGAAA | 5' TCGTTCGCCAAATAGTGAGA |
| *sem3e* | g5331.t1 | 5' CGCCTCATGGCTTATTGTTT | 5' CTCTTCCAGCACCTCCAAAG |
| *bmp4* | g189.t1 | 5' TCTGCATCTGAACCGAACAC | 5' TCATGACCGAAAGTGACCAA |
| *smad3* | g3356.t1 | 5' AGCTGGACGAGTTGGAGAAA | 5' TCACATGAGGGAGACCCTTC |
| *at233* | g9311.t1 | 5' GAGCCTGCTCAGGCATAGAT | 5' GCGACACATTGCTTTTGAGA |
| *cahz* | g8816.t1 | 5' AGTGACCTTCACGGATGAGG | 5' TGTGCTCAGACCCTTTGTTG |
| *cah2* | g3019.t1 | 5' GGCTCAGAGCACACAGTTGA | 5' GGAGCCATCGTATGTCCAGT |
| *mb* | g8414.t1 | 5' CACCCTGACACCCAGAAACT | 5' TTTGCCTTGAGGATCTCACC |
| *cah6* | g456.t1 | 5' AACCTGGGTCATCCAATGAA | 5' TGAGCTCGTTTTCGTGTTTG |
| *slc9a3* | g3908.t1 | 5' TTGCTGTGTTCGAAGAGGTG | 5' CCAGAGACACAAAGGCATCA |
| *slc4a1* | g12078.t1 | 5' TGTAGGCCTGTCCATCATCA | 5' TATGCGATCCCACAGTTGAA |
